# Targeting MYC effector functions in pancreatic cancer by inhibiting the ATPase RUVBL1/2

**DOI:** 10.1136/gutjnl-2023-331519

**Published:** 2024-05-31

**Authors:** Markus Vogt, Nevenka Dudvarski Stankovic, Yiliam Cruz Garcia, Julia Hofstetter, Katharina Schneider, Filiz Kuybu, Theresa Hauck, Bikash Adhikari, Anton Hamann, Yamila Rocca, Lara Grysczyk, Benedikt Martin, Anneli Gebhardt-Wolf, Armin Wiegering, Markus Diefenbacher, Georg Gasteiger, Stefan Knapp, Dieter Saur, Martin Eilers, Mathias Rosenfeldt, Florian Erhard, Seychelle M Vos, Elmar Wolf

**Affiliations:** 1 Cancer Systems Biology Group, Chair of Biochemistry and Molecular Biology, Theodor Boveri Institute, University of Würzburg, Würzburg, Germany; 2 Institute of Biochemistry, University of Kiel, Kiel, Germany; 3 Department of Biology, Massachusetts Institute of Technology, Cambridge, Massachusetts, USA; 4 Institute for Pharmaceutical Chemistry, Goethe-University Frankfurt, Frankfurt am Main, Germany; 5 Max Planck Research Group and Institute of Systems Immunology, University of Würzburg, Würzburg, Germany; 6 Chair of Biochemistry and Molecular Biology, Theodor Boveri Institute, University of Würzburg, Würzburg, Germany; 7 Department of General, Visceral, Transplantation, Vascular and Pediatric Surgery, University Hospital Würzburg, Würzburg, Germany; 8 Comprehensive Pneumology Center (CPC)/Institute of Lung Health and Immunity (LHI), Helmholtz Munich, Member of the German Center for Lung Research (DZL/CPC-M), Munich, Germany; 9 Ludwig-Maximilian-Universität München (LMU), Munich, Germany; 10 Institute of Translational Cancer Research, TUM School of Medicine and Health, Munich, Germany; 11 Institute of Pathology, University of Würzburg, Würzburg, Germany; 12 Computational Systems Virology and Bioinformatics, Institute for Virology and Immunobiology, University of Würzburg, Würzburg, Germany

**Keywords:** PANCREATIC CANCER, GENE REGULATION, ONCOGENES, IMMUNOTHERAPY, GENE TARGETING

## Abstract

**Objective:**

The hallmark oncogene MYC drives the progression of most tumours, but direct inhibition of MYC by a small-molecule drug has not reached clinical testing. MYC is a transcription factor that depends on several binding partners to function. We therefore explored the possibility of targeting MYC via its interactome in pancreatic ductal adenocarcinoma (PDAC).

**Design:**

To identify the most suitable targets among all MYC binding partners, we constructed a targeted shRNA library and performed screens in cultured PDAC cells and tumours in mice.

**Results:**

Unexpectedly, many MYC binding partners were found to be important for cultured PDAC cells but dispensable in vivo. However, some were also essential for tumours in their natural environment and, among these, the ATPases RUVBL1 and RUVBL2 ranked first. Degradation of RUVBL1 by the auxin-degron system led to the arrest of cultured PDAC cells but not untransformed cells and to complete tumour regression in mice, which was preceded by immune cell infiltration. Mechanistically, RUVBL1 was required for MYC to establish oncogenic and immunoevasive gene expression identifying the RUVBL1/2 complex as a druggable vulnerability in MYC-driven cancer.

**Conclusion:**

One implication of our study is that PDAC cell dependencies are strongly influenced by the environment, so genetic screens should be performed in vitro and in vivo. Moreover, the auxin-degron system can be applied in a PDAC model, allowing target validation in living mice. Finally, by revealing the nuclear functions of the RUVBL1/2 complex, our study presents a pharmaceutical strategy to render pancreatic cancers potentially susceptible to immunotherapy.

**WHAT IS ALREADY KNOWN ON THIS TOPIC**

- Dozens of MYC binding partners have been identified, but no systematic analysis has investigated their suitability as cancer targets in a real tumour context. Our screening hit RUVBL1 is a case in point. While it has been shown that RUVBL1 is essential for MYC-mediated anchorage-independent growth of fibroblasts, it remained unclear whether RUVBL1 is an essential effector protein of MYC in established pancreatic ductal adenocarcinoma (PDAC) in vivo and thus a viable therapeutic target.

**WHAT THIS STUDY ADDS**

- Our work has shown that only a few MYC binding partners are essential for PDAC progression in mice. One of them, RUVBL1, is required for the activation of MYC-driven gene expression and immune evasion. Acute depletion of RUVBL1 by the auxin-degron system induced rapid eradication of PDAC tumours, accompanied by a significant infiltration of CD3-positive immune cells.

**HOW THIS STUDY MIGHT AFFECT RESEARCH, PRACTICE OR POLICY**

- Advanced PDAC remains incurable despite recent advances in targeted therapies and immunotherapy. The identification of RUVBL1 as a target in MYC-driven PDAC tumours and our observation that RUVBL1 depletion sensitises to immune checkpoint inhibitors opens a therapeutic strategy, as this AAA ATPase could be considered druggable by small-molecule inhibitors.

## Introduction

Advanced solid tumours are still incurable in many cases, despite the successful development of targeted cancer therapies in recent years.^1^ An important example where only limited therapeutic progress has been made is pancreatic ductal adenocarcinoma (PDAC), a cancer that derives from the epithelial exocrine pancreas. Patients with PDAC have a median survival of less than 1 year,^2^ resulting in 530 000 deaths per year worldwide.

The main limitation of targeted cancer therapy, in general and particularly in PDAC, is its narrow applicability. Indeed, each targeted therapy is designed for a specific subset of patients whose tumours have specific genetic profiles.^3^ While the availability of a targeted therapy is an attractive therapeutic option for this particular patient subgroup, the corresponding genetic mutation is often rare, so the number of patients who can benefit is small. This conundrum also applies to immunotherapies, where only patients with an immunologically active tumour microenvironment benefit from immune checkpoint blockade.^4^ To date, it has proven impossible to develop a universally applicable targeted cancer therapy by inhibiting common oncogenes.

An important example of oncogenes with universal significance is the family of MYC transcription factors, which comprises c-MYC (called MYC hereafter), NMYC and LMYC.^5 6^ Several lines of experimental and clinical research suggest that MYC should be an outstanding cancer target. Most tumours have deregulated MYC expression caused by various mechanisms including somatic gene amplifications and translocations, mutations that increase MYC protein stability and alterations in upstream pathways that enhance MYC transcription.^7^ The link between high MYC expression and tumorigenesis is causative, since exogenous expression of MYC in mouse models initiates^8^ or accelerates^9^ tumour development. Importantly, deregulated MYC expression drives tumour initiation and renders established tumours ‘addicted’ to it, since silencing exogenous MYC irreversibly eradicates tumour cells.^10–14^ Moreover, systemic expression of a dominant negative allele of MYC, called Omomyc, induced regression of established murine tumours but was tolerated by normal cells,^15^ suggesting the existence of a therapeutic window for the pharmaceutical inactivation of MYC. Strikingly, periodic inhibition of endogenous MYC by Omomyc permanently prevented tumour progression in mice without any sign of resistance.^16^ Recombinant Omomyc showed therapeutic efficacy in preclinical models of lung cancer^17^ and has been tested for safety in a phase I clinical trial (NCT04808362).^18^

Clinical testing of an MYC-derived therapeutic protein is exciting, but it might be difficult to deliver macromolecules to tumours in organs such as the brain and pancreas.^19^ Indeed, protein-based drugs have more limited drug-delivery properties compared with small-molecule drugs, but so far no small-molecule inhibitor of MYC has advanced to clinical testing for various reasons. MYC lacks catalytic activity that can be easily inhibited; instead, MYC functions by binding other proteins and DNA. The DNA-binding domain of MYC is formed by extensive interactions with its partner MAX^20^ and is structurally similar to the corresponding domains of other basic helix-loop-helix transcription factors. The transactivation domain of MYC is intrinsically disordered. These structural features impede the development of small-molecule drugs that directly inhibit MYC.

An attractive alternative to inhibiting MYC directly is targeting a binding partner that is a better drug target than MYC itself. MYC’s DNA binding and transactivation activities both depend on MYC binding partners, as has been explicitly shown for MAX, WDR5, PAF, SPT5 and TRRAP.^21–28^ However, MYC has broad affinity to other proteins, and dozens of putative binding partners have been identified by systematic proteomics methods.^28–31^ It is currently unclear which of them are essential for MYC function and oncogenic growth in PDAC. We therefore used a targeted shRNA library to analyse the dependence of pancreatic cancer cells on 91 MYC binding partners, in vitro and in vivo. Our genetic screens revealed that among the most essential MYC binding partners were RUVBL1 and RUVBL2, which are AAA ATPases that form heteromeric complexes. We observed that oncogenic MYC expression renders cells dependent on the activity of the RUVBL1/2 complex and that acute depletion or inhibition of RUVBL1 provokes immune infiltration and eradicates pancreatic tumours in mice.

## Results

### Genetic dropout screens reveal differential dependence on MYC binding partners in PDAC in vitro and in vivo

To assess the essentiality of MYC binding partners in PDAC, we generated a doxycycline-inducible shRNA library (containing five shRNAs per gene and 18 non-targeting control (NTC) shRNAs)^32^ against 91 MYC binding partners identified in quantitative mass spectrometry (MS) experiments of natively isolated MYC complexes. Many of the candidates have been identified multiple times in additional published interaction studies^28–31^ (online supplemental table 1). PCR amplification of the library (online supplemental figure S1A,B) followed by sequencing identified all 478 expected shRNAs. The abundance of 93.3% of the shRNAs differed by less than 10-fold, verifying their even distribution within the library (online supplemental figure S1C).^33^

10.1136/gutjnl-2023-331519.supp1 Supplementary data

10.1136/gutjnl-2023-331519.supp2 Supplementary data

**Figure 1:**
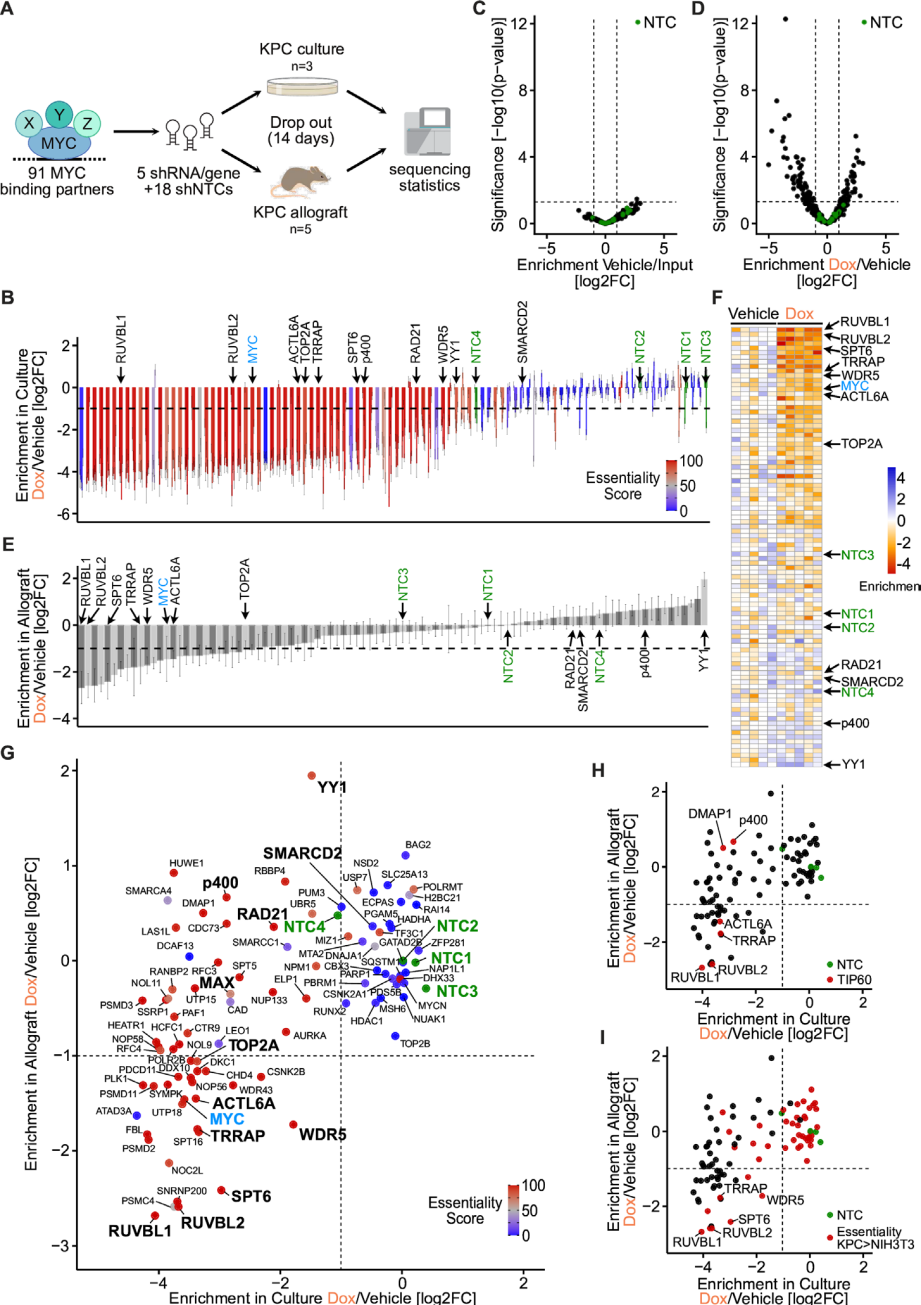
Genetic dropout screens reveal differential dependence on MYC binding partners in pancreatic ductal adenocarcinoma (PDAC) in vitro and in vivo. (A) Schematic of the in vitro (KPC culture) and in vivo (KPC allograft) dropout screens for 91 MYC binding partners. (B) Waterfall plot of the in vitro screen in KPC cells showing the doxycycline (Dox)-induced changes in abundance of 478 shRNAs (log2FC±SEM, n=3). shRNAs are grouped by target gene, sorted by the median change for the five shRNAs. Selected genes of interest, including MYC, are marked. The essentiality score is the percentage of human cancer cell lines in the DepMap portal that depend on each gene for viability. NTC1–4 correspond to groups of four or five non-targeting control shRNAs. (C) Volcano plot of the in vivo screen showing changes in abundance (enrichment) for 460 targeting shRNAs (black dots) and 18 non-targeting shRNAs (green dots), between five tumours from mice fed standard chow (here, ‘Vehicle’) and transplanted KPC cells (Input). Wald test p values for five mice. (D) Volcano plot of the in vivo screen showing changes in abundance (enrichment) for 460 targeting shRNAs (black dots) and 18 non-targeting shRNAs (green dots), between five tumours from mice fed doxycycline-containing chow (Dox) and five tumours from mice fed standard chow (Vehicle). Wald test p values. (E) Waterfall plot of the in vivo screen comparing integrated shRNA abundance in tumours of five mice fed doxycycline-containing chow (Dox) and five mice fed standard chow (Vehicle). Bars represent the median log2FC for five shRNAs per gene, and error bars represent the SEM. (F) Heatmap of the in vivo screen. The chromatic scale indicates the median change in integrated shRNA abundance (log2FC) per gene relative to vehicle-treated mice. Each column corresponds to one mouse fed either standard chow (Vehicle) or doxycycline-containing chow (Dox). Genes are sorted by the median change for five Dox-treated mice. (G) Scatter plot comparing the results of the in vitro (cultured KPC cells) and in vivo (allografted KPC cells) screens. Dots represent the median change (log2FC) of five shRNAs per gene and are colour coded according to the essentiality score. NTC1–4, groups of four or five NTC shRNAs. (H) Scatter plot as in panel G. *Red*, TIP60 complex components. *Green*, NTC shRNAs. (I) Scatter plot as in panel G. *Red*, genes that are more essential in KPC cells than in NIH3T3 cells (values are reported in online supplemental table 2). *Green*, NTC shRNAs. See also online supplemental figure S1.

The shRNA library was used for genetic dropout screening in both in vitro and in vivo PDAC models (figure 1A). First, the oncogenic potential of the 91 MYC binding partners was tested in the murine KPC cell line harbouring oncogenic mutations in KRAS and p53,^34^ because this cell line engrafts in C57BL/6J mice and forms MYC-dependent tumours.^35^ KPC cells were transduced with the library, then split and cultured without or with doxycycline to activate shRNA expression. After 14 days, genomic DNA was isolated and sequenced to identify integrated shRNAs in the two treatment groups. This analysis showed that for each shRNA the changes were similar among three replicates, and for each MYC binding partner the changes were similar among the five shRNAs, documenting the robustness of the screening system (figure 1B). Furthermore, it demonstrated that expression of shRNAs against 61 of the targets led to an at least twofold dropout, suggesting their essentiality for KPC cell growth (online supplemental table 2). We wondered whether the dependence of KPC cells on specific MYC binding partners differs in cultured cancer cells of other tumour types. When we calculated an ‘essentiality score’ (percentage of 1086 cancer cell lines in DepMap for which a gene is essential),^36^ we observed that 54 of the 61 partners were essential in >50% of all tested cancer cell lines. Only the candidate ATAD3A, which also exerts mitochondrial functions, shows strong deviations from the DepMap database, which may be related to synthetic effects with the doxycycline used in our screen.^37^ Taken together, these results suggest that the viability of cultured KPC cells depends on the majority of MYC binding partners, of which most are also essential for cultured cells of other tumour types.

10.1136/gutjnl-2023-331519.supp3 Supplementary data

Since conditions in cell culture are fundamentally different from those in a living organism, we investigated whether the dependence of pancreatic cancer cells on MYC binding partners is different in a tumour context. We set up an in vivo genetic screen using shRNA-transduced KPC cells orthotopically engrafted into the pancreas of immunocompetent mice (figure 1A). A first experiment revealed that an injection of 100 000 cells was needed to achieve uniform representation of the library (online supplemental figure S1C). We therefore engrafted 10 mice with 100 000 cells each, induced shRNA expression by doxycycline in five of the animals and analysed integrated shRNA abundance in tumours after 14 days.

First, we compared the abundance of integrated shRNA in the five tumours of the untreated mice with that in the originally transduced KPC cells (figure 1C). No shRNA was found to be significantly depleted in tumours, confirming that shRNA expression is not induced in the absence of doxycycline. In a comparison between doxycycline-treated and untreated mice, 70 of the 460 targeting shRNAs were significantly depleted (log2 fold change (log2FC) <−1, p<0.05), while the abundance of the 18 NTC shRNAs was unchanged (figure 1D). When we examined the median shRNA change per gene, we observed that only 26 MYC binding partners were significantly depleted (figure 1E,F).

To understand how the dependency of KPC cells differs between the in vitro and in vivo conditions, we compared the doxycycline-induced change in all MYC binding partners in a scatter plot (figure 1G; see also online supplemental table 2). This analysis revealed that 31 MYC binding partners, such as the SMARCD2 subunit of the SWI/SNF chromatin remodelling complex, are not essential in either condition. Furthermore, 34 MYC binding partners were found to be essential in vitro but dispensable in vivo. This group contained many proteins that are considered common essential genes in DepMap, including proteins that are considered oncogenic targets such as the transcription factors YY1^38^ and p400,^39^ and the cohesin complex member RAD21.^40^ Remarkably, the constitutive binding partner of MYC’s DNA binding domain, MAX,^5 41^ was also not essential in vivo. We hypothesise that partial depletion of MAX is neutral in pancreatic tumours, since MAX also forms repressive homodimers and heterodimers that bind to similar DNA motifs and compete with the gene-activating capacity of the MYC/MAX heterodimers. This observation is also consistent with the dominant-negative function of Omomyc, which shifts MAX from activating MYC/MAX to transcriptionally inactive Omomyc/Max dimers.^15 16 42 43^

Finally, 27 genes were found to be essential both in vitro and in vivo. Among these proteins are MYC itself and its well-characterised partners WDR5,^23^ TOP2A^44^ and SPT6.^28^ Strikingly, this group also included four members of the TIP60 complex, namely RUVBL1, RUVBL2, TRRAP and ACTL6A (figure 1H).

To determine if there are MYC binding partners that are essential for PDAC tumours but dispensable for non-cancer cells, we repeated the dropout screen in the NIH3T3 fibroblast cell line. This analysis showed that 51 MYC binding partners are essential for the growth of these untransformed cells (log2FC <−1) (online supplemental table 2). A comparison with KPC cells revealed that only RUVBL1, RUVBL2, TRRAP, SPT6 and WDR5 were essential in pancreatic tumours and more important for KPC cells than fibroblasts (Δlog2FC<0, figure 1I). We concluded that (1) the dependencies of pancreatic cancer cells on MYC binding partners differed in vitro and in vivo and that (2) five candidates were less essential for fibroblast than for PDAC tumours.

### Expression of MYC and RUVBL1 correlate in PDAC and high levels are associated with aggressive tumours

Next, we wanted to find out which MYC effector proteins are highly expressed in PDAC tumours with high MYC activity, but show low expression in tumours with low MYC levels and in normal tissue. For this purpose, we first analysed the co-expression of all 91 candidates with MYC in tumours from patients with PDAC collected in the curated TCGA database (n=159).^45^ We correlated the RNA expression of MYC target genes (as a proxy for MYC activity) with the RNA expression of every candidate in PDAC primary tumour samples (online supplemental figure S2A, online supplemental table 3). The expression of 25 MYC binding partners correlated strongly with MYC levels (Pearson’s correlation coefficient >0.5). We then compared the expression of all MYC binding partners in PDAC tumours to their expression in healthy pancreatic tissue (n=4, online supplemental figure S2B, online supplemental table 3) revealing that 25 MYC binding partners are overexpressed in PDAC samples compared with healthy tissue (log2FC>0.3).

10.1136/gutjnl-2023-331519.supp4 Supplementary data

**Figure 2:**
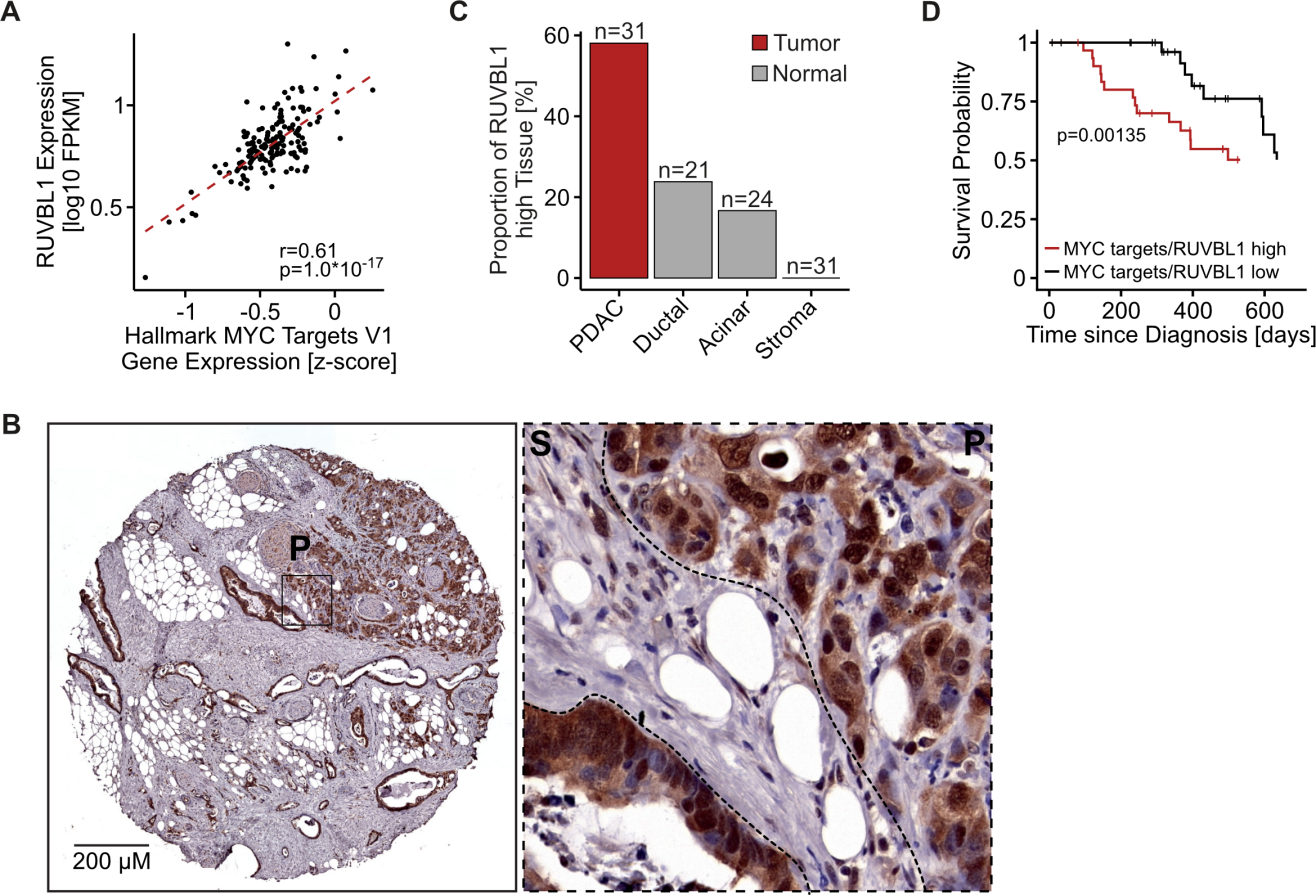
Expression of MYC and RUVBL1 correlate in pancreatic ductal adenocarcinoma (PDAC) and high levels are associated with aggressive tumours. (A) Scatter plot comparing expression of RUVBL1 and MYC target gene expression (mean of all HALLMARK MYC TARGET V1 genes after scaling expression (FPKM) across all TCGA patients) in patients with human PDAC from the TCGA database (r, Pearson’s correlation coefficient; p value, unpaired t-test, n=159). (B) Exemplary immunohistochemistry of RUVBL1 from a tissue core from a tissue microarray containing 31 individual human PDAC specimens. A section with high RUVBL1 expression and a zoom-in with PDAC (P) and stromal tissue (S) are shown (scale: 200 µm). The panel is also shown as part of online supplemental figure S2C. (C) Quantification of RUVBL1 expression in a tissue microarray containing 31 sections of human PDAC specimens and 24 sections of benign acinar tissue as in panel B. RUVBL1 expression was scored as negative, low, medium and high in PDAC, adjacent stroma and non-malignant ductal and acinar tissue. The ratio of tissues with high RUVBL1 expression is shown. n, sample size. (D) Kaplan-Meier survival curves for patients with PDAC stratified into groups of low and high expression of RUVBL1 and MYC target genes (mean of all MYC TARGET V1 genes after scaling expression (FPKM) across all TCGA patients). P value, log-rank test. See also online supplemental figure S2.

**Figure 3:**
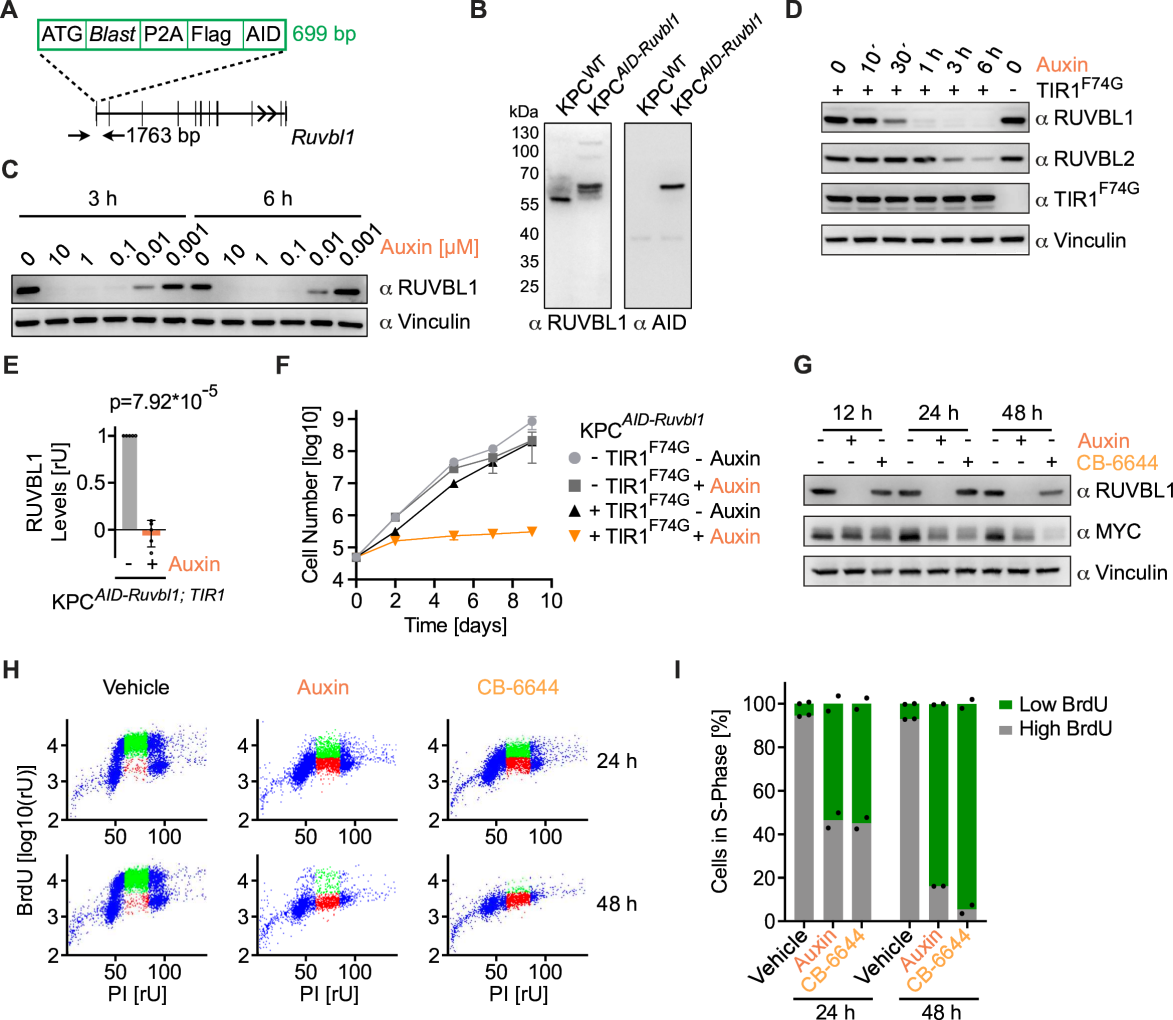
RUVBL1 is essential for DNA replication and growth of pancreatic cancer cells. (A) *Ruvbl1* knock-in strategy for auxin-inducible degron (AID) tagging showing the elements of the knock-in cassette and the architecture of *Ruvbl1*. Arrows indicate the position of primers used to identify recombined cell clones by PCR (see online supplemental figure S3B). (B) Immunoblots of wild-type KPC and KPC*^AID-Ruvbl1^* cell lysates probed with antibodies against RUVBL1 or the AID tag. (C) Immunoblot of KPC*^AID-Ruvbl1; TIR1^* cells treated with various concentrations of auxin for 3 or 6 hours. Vinculin, loading control. (D) Immunoblot of KPC*^AID-Ruvbl1; TIR1^* cells treated with 1 µM auxin over time. TIR1^F74G^ was detected with an antibody against the MYC tag. Vinculin, loading control. (E) Quantification of immunoblots of RUVBL1 after 6 hours of 1 µM auxin treatment (n=5, mean±SD, unpaired t-test). rU, relative units. (F) Logarithmic growth curve of KPC*^AID-Ruvbl1^* cells expressing or not expressing TIR1^F74G^. Cells were treated daily with 1 µM auxin or vehicle, and growth was followed for 9 days in biological triplicates (n=3, mean±SD). (G) Immunoblot of KPC*^AID-Ruvbl1; TIR1^* cells treated with 1 µM auxin or 1 µM CB-6644 for indicated time points. Vinculin, loading control. (H) BrdU-PI flow cytometry scatter plots of KPC*^AID-Ruvbl1; TIR1^* cells after treatment with 1 µM auxin or 1 µM CB-6644 for 24 or 48 hours. Cells were labelled with BrdU for 1 hour. (I) Quantification of S-phase cells with high or low BrdU incorporation as shown in panel H. The experiment was performed in biological duplicates (n=2, mean). See also online supplemental figure S3.

The only MYC binding partners that (1) showed depletion in the in vivo screen, (2) had a good correlation between their expression and MYC activity in PDAC tumours (figure 2A) and (3) were overexpressed in PDAC tumours compared with healthy pancreatic tissue were the proteins RUVBL1 and RUVBL2, with RUVBL1 scoring the highest. We validated increased RUVBL1 expression in patients with human cancer at the protein level using an in-house tissue microarray (TMA) containing PDAC tumour, benign acinar and benign ductal tissue specimens stained for RUVBL1 (figure 2B,C, online supplemental figure S2C,D). Strikingly, patients with high levels of MYC and RUVBL1 show significantly reduced overall survival compared with patients with low expression (figure 2D, online supplemental figure S2E). Interestingly, RUVBL1 expression is higher in basal and undifferentiated tumours than in glandular and differentiated tumours, respectively (online supplemental figure S2F,G). We concluded that patients with PDAC with elevated MYC expression also overexpress RUVBL1, and that tumours with the highest expression of both proteins are the most aggressive.

### RUVBL1 is essential for DNA replication and growth of pancreatic cancer cells

RUVBL1 and RUVBL2 are AAA ATPases that form heteromeric hexamers or dodecamers (online supplemental figure S3A).^46^ In cells, they are subunits of several multiprotein complexes, such as the R2TP/PAQosome chaperone complex, the histone acetyltransferase complex TIP60 and the chromatin remodelling complex INO80. Moreover, recently their role in RNA polymerase II (RNAPII) clustering has been described.^47^ We aimed to establish a targeted protein depletion system for the rapid degradation of RUVBL1 to study the direct oncogenic function of the RUVBL1/2 complex in PDAC. For this purpose, we inserted the auxin-inducible degron (AID) sequence into the *Ruvbl1* locus in KPC cells (figure 3A, online supplemental figure S3B,C). Immunoblotting confirmed the successful fusion of the AID tag with RUVBL1 and the lack of expression of wild-type RUVBL1 (figure 3B). We then stably expressed in these cells the plant-derived E3 ligase TIR1^F74G^ that, in the presence of auxin (5-phenyl-1H-indole-3-acetic acid), enables the degradation of AID-tagged proteins at low auxin concentrations.^48^ Accordingly, we observed degradation of RUVBL1 in KPC*^AID-Ruvbl1; TIR1^* cells at nanomolar concentrations and complete depletion at 1 µM auxin (figure 3C). Strikingly, incubation of KPC*^AID-Ruvbl1; TIR1^* cells with auxin for 6 hours decreased RUVBL1 to undetectable levels (figure 3D,E).

Auxin-mediated depletion of RUVBL1 in KPC*^AID-Ruvbl1; TIR1^* cells substantially stopped cell growth, while auxin had no impact on the growth of KPC*^AID-Ruvbl1^* cells without TIR1^F74G^ (figure 3F). To understand why the cells arrested on RUVBL1 depletion, we analysed the cell cycle distribution by BrdU-PI flow cytometry (figure 3G–I, online supplemental figure S3D). Auxin-mediated depletion of RUVBL1 reduced the number of BrdU-positive cells at 24 hours and completely prevented any BrdU incorporation after 48 hours. Similar effects were observed with CB-6644, an allosteric RUVBL1/2 inhibitor^49^ (figure 3G–I, online supplemental figure S3D). These results suggest that RUVBL1/2 complex formation or catalytic activity is crucial for the growth-promoting function of RUVBL1. Moreover, the observed arrest in S-phase on depletion or inhibition of RUVBL1 was comparable to what was described on silencing MYC in pancreatic cancer cells.^35^

### RUVBL1 redirects transcription from immune genes to growth genes

To determine if RUVBL1 directly influences transcription, we combined auxin-mediated depletion with SLAM-seq, a method to identify new transcripts by 4sU labelling. We treated KPC*^AID-Ruvbl1; TIR1^* cells with auxin for 3 or 15 hours to deplete RUVBL1 and labelled newly synthesised transcripts with 4sU for 2 hours (online supplemental figure S4A). The transcriptional consequences of 3 hours of RUVBL1 depletion were similar to those of 15 hours, but the overall impact was slightly weaker with some transcripts being affected differently (online supplemental figure S4B, online supplemental table 4).

10.1136/gutjnl-2023-331519.supp5 Supplementary data

**Figure 4:**
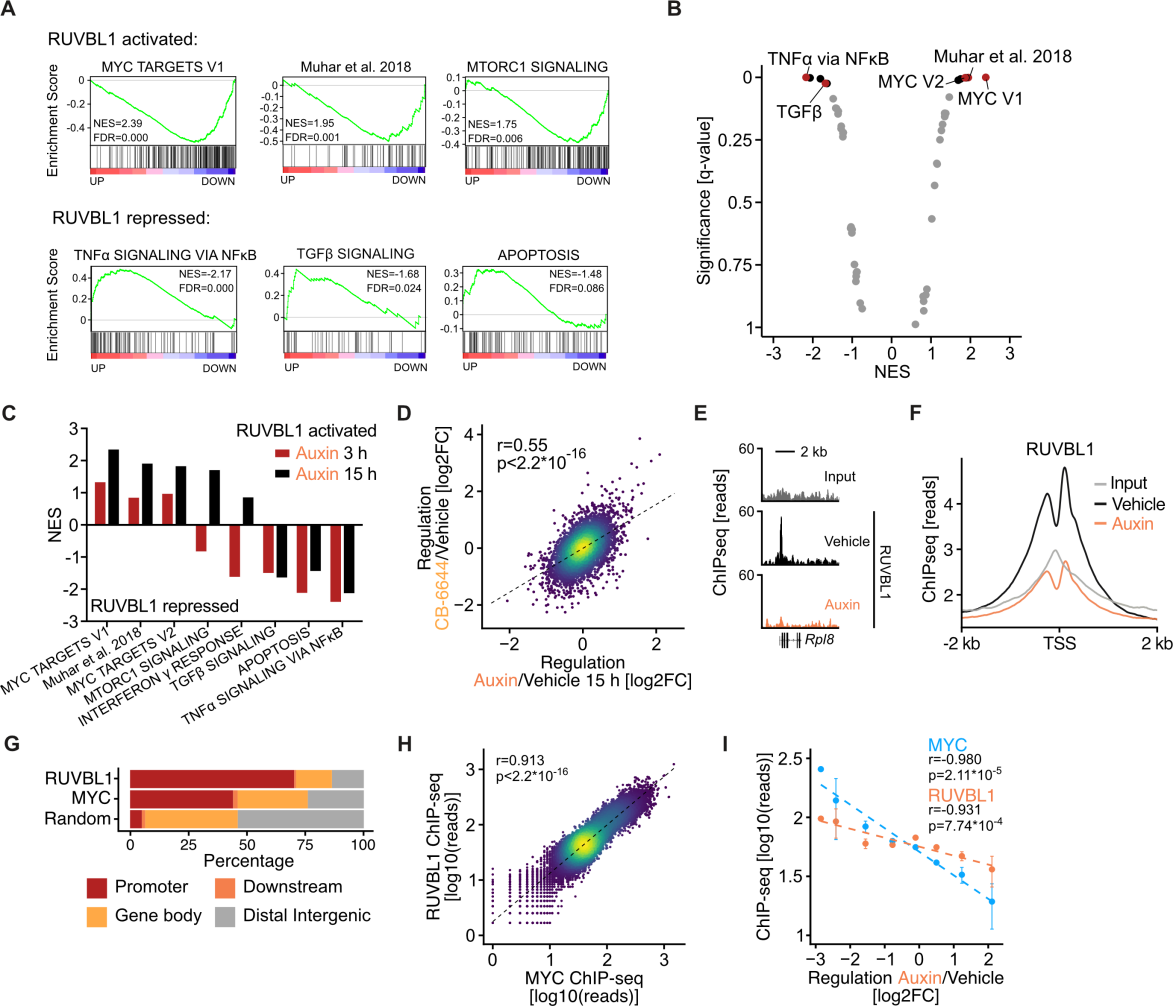
RUVBL1 redirects transcription from immune genes to growth genes. (A) Gene set enrichment analysis (GSEA) plots of selected RUVBL1-activated and RUVBL1-repressed gene sets. GSEA was performed on SLAM-seq data of KPC*^AID-RUVBL1; TIR1^* cells. Cells were treated with 1 µM auxin or DMSO (Vehicle) for 15 hours, followed by 800 µM 4sU for 2 hours. The normalised enrichment score (NES) is positive for gene sets activated by and negative for those repressed by RUVBL1 (FDR, false discovery rate). (B) GSEA of KPC*^AID-Ruvbl1; TIR1^* cells treated with 1 µM auxin or DMSO for 15 hours and 800 µM 4sU. The NES and the q-value are shown for all enriched gene sets (positive NES: gene sets activated by RUVBL1, negative NES: gene sets repressed by RUVBL1). (C) NES values for different hallmark gene sets and a gene set containing primary MYC targets defined in Muhar *et al*,^50^ compared between the 3 and 15-hour durations of RUVBL1 depletion. (D) Scatter plot comparing gene regulation after auxin-induced degradation and CB-6644 inhibition of RUVBL1. KPC*^AID-Ruvbl1; TIR1^* cells were treated with 1 µM auxin for 15 hours (n=3) or 1 µM CB-6644 for 24 hours (n=3). Gene expression was analysed by SLAM-seq. Changes (log2FC) in total RNA versus DMSO (vehicle)-treated cells are shown (r, Pearson’s correlation coefficient; p value, unpaired t-test). (E) Genome browser track of RUVBL1 chromatin immune precipitation (ChIP)-seq signal in KPC*^AID-Ruvbl1; TIR1^* cells treated with vehicle or 1 µM auxin for 15 hours. Binding to the *Rpl8* gene is shown as spike-normalised reads and compared with the input signal as control. (F) Density plots of RUVBL1 ChIP-seq signals around transcription start sites (TSS). Averaged binding (RPKM) of RUVBL1 in KPC*^AID-Ruvbl1; TIR1^* cells treated with DMSO (vehicle) or 1 µM auxin for 15 hours compared with the input signal. (G) Bar graph of the genomic distribution of RUVBL1 and MYC peaks. Chromatin binding of RUVBL1 and MYC was analysed by ChIP-seq and was compared with a set of random genomic intervals in promoters (TSS− 3000 bp to TSS+ 3000 bp), gene bodies (TSS+ 3000 bp to transcription end site (TES)), regions downstream of genes (TES to TES+ 2000 bp) and intergenic regions. (H) Scatter plot comparing the promoter occupancy of MYC and RUVBL1 (spike-normalised reads) as measured by ChIP-seq. r, Pearson’s correlation coefficient; p value, unpaired t-test. (I) Bin plot comparing gene regulation due to acute RUVBL1 depletion with RUVBL1 and MYC binding (spike-normalised reads) at gene promoters. Genes were binned into eight equally distant bins of gene regulation on 15 hours of RUVBL1 depletion (‘Regulation’). Mean regulation per bin was plotted against mean promoter occupancy by MYC and RUVBL1 in unperturbed KPC*^AID-Ruvbl1; TIR1^* cells (mean±SEM). r, Pearson’s correlation coefficient; p value, unpaired t-test. NFkB, nuclear factor kappa B; TGFβ, transforming growth factor-beta; TNFα, tumour necrosis factor alpha. See also online supplemental figure S4.

We used gene set enrichment analysis to analyse RUVBL1-mediated transcriptional consequences and the underlying kinetics. Strikingly, transcripts of known MYC target genes were downregulated (ie, activated by RUVBL1) at both time points and were overall the most strongly affected genes (figure 4A–C, online supplemental table 5). This group of genes includes a set of MYC-induced transcripts identified earlier, in an AID depletion system for MYC, as primary MYC target genes.^50^ The most upregulated genes (hence repressed by RUVBL1) after acute RUVBL1 depletion encode proteins mediating immune signalling, such as components of the tumour necrosis factor alpha, transforming growth factor-beta and interferon-γ-signalling pathways (figure 4A–C); these genes have already been shown to be repressed by MYC in various systems.^12–14 35 51^ Treatment of KPC cells with the RUVBL1/2 complex inhibitor CB-6644 for 24 hours induced similar transcriptional changes as did RUVBL1 depletion (figure 4D, online supplemental figure S4C). To test whether activation of MYC target genes and repression of immune genes is a general function of RUVBL1 in pancreatic cancer cells, we treated five further murine PDAC lines with different genetic backgrounds with CB-6644 for 24 hours and analysed gene expression by RNA sequencing. Strikingly, we observed a robust upregulation of interferon signalling and downregulation of MYC target genes in all cell lines tested (online supplemental figure S4D,E).

10.1136/gutjnl-2023-331519.supp6 Supplementary data

We wondered if RUVBL1 regulates MYC target genes by binding to their promoters. Therefore, we studied RUVBL1 and MYC binding to chromatin globally in KPC*^AID-Ruvbl1; TIR1^* cells treated with vehicle or auxin by chromatin immune precipitation (ChIP) followed by sequencing. In vehicle-treated cells, RUVBL1 occupied 10 016 genomic sites, of which 7386 were in RNAPII promoters (online supplemental figure S4F). Auxin-mediated depletion of RUVBL1 drastically reduced the number of RUVBL1-occupied sites to 805 and brought the signals at promoters to background levels (figure 4E,F). MYC occupied 15 991 genomic sites, of which 7906 were in promoters (online supplemental figure S4F). Since the absolute number of peaks strongly depends on the chosen peak calling algorithm and thresholds (online supplemental figure S4G), and since both MYC and RUVBL1 show a strong promoter preference (figure 4G), we quantified the occupancy of both proteins at all annotated promoters and observed a striking correlation (r=0.913, figure 4H, online supplemental figure S4H).

We wondered if promoter occupancy of MYC and RUVBL1 correlates with the transcription changes due to acute RUVBL1 depletion. We sorted all transcripts identified in the SLAM-seq experiment according to their regulation on RUVBL1 depletion and compared this ranking to the extent of promoter occupancy by MYC and RUVBL1 in a bin plot (figure 4I). These analyses revealed that genes activated by RUVBL1 have higher promoter occupancy of both MYC and RUVBL1 than non-regulated and repressed genes. We concluded that RUVBL1 and MYC co-occupy thousands of promoters in pancreatic cancer cells and that depletion or inhibition of RUVBL1 leads to their downregulation.

### RUVBL1/2 complex is an essential cofactor of MYC

**Figure 5:**
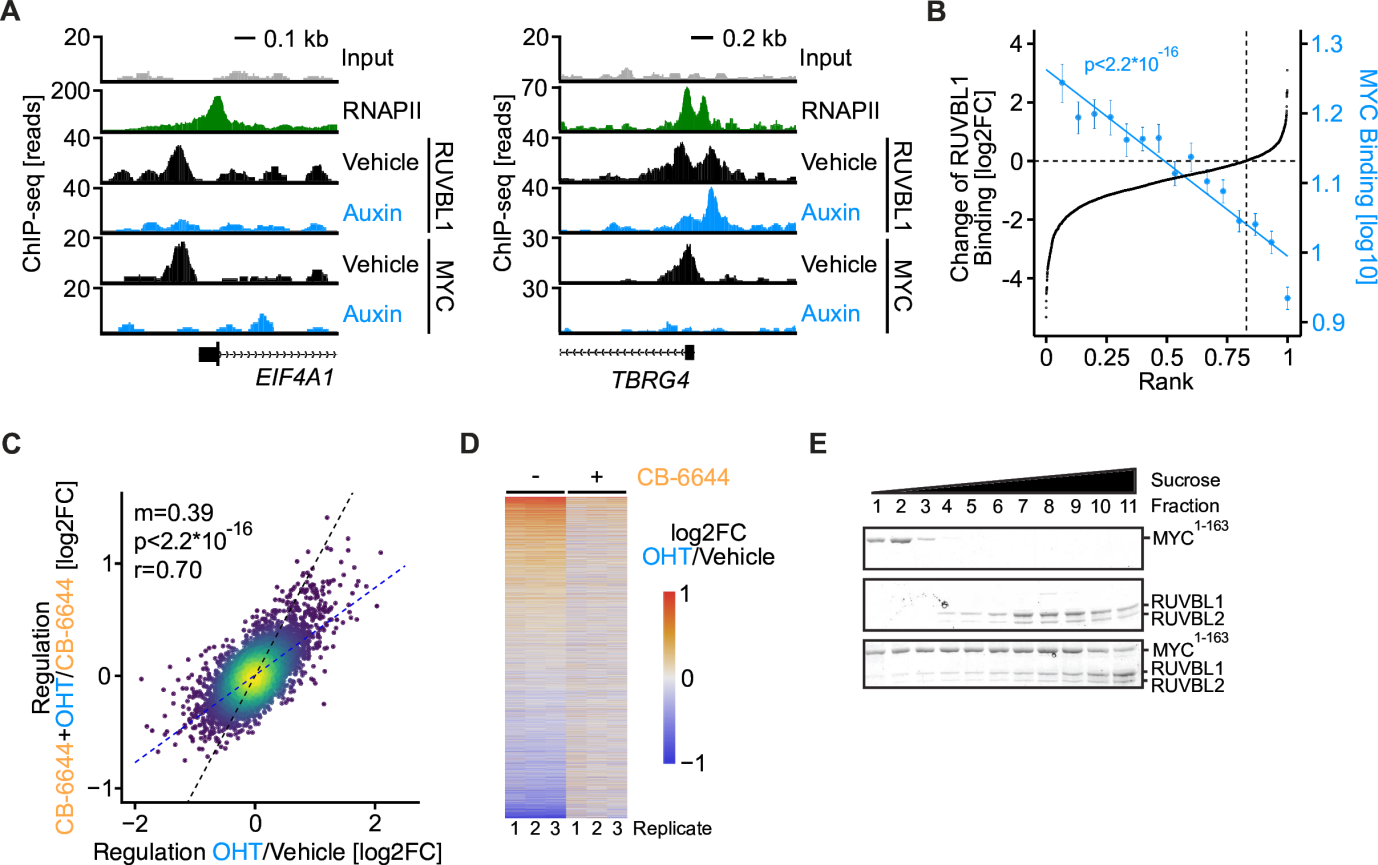
RUVBL1 is an essential cofactor of MYC. (A) Genome browser tracks of RUVBL1 and MYC chromatin immune precipitation (ChIP)-seq signal in A375*^MYC-AID^* cells treated with 1 µM auxin for 3 hours or vehicle control. RNA polymerase II (RNAPII) tracks of U2OS cells (GSE162264) are shown. Binding to the EIF4A1 and TBRG4 genes is shown as spike-normalised reads and compared with the input signal as control. In the TBRG4 promoter, one of two RUVBL1 peaks located at an MYC-negative region does not decrease on auxin-mediated depletion of MYC. (B) Rank plot of RUVBL1 and MYC ChIP-seq signal in A375*^MYC-AID^* cells treated with 1 µM auxin for 3 hours or vehicle control. All MYC and RUVBL1-bound promoters are sorted for decrease in RUVBL1 chromatin binding on acute MYC depletion and plotted as log2FC (y-axis, black). Mean MYC binding is plotted for 15 equally sized bins containing the same genes (y-axis, blue). (C) Scatter plot of SLAM-seq data comparing MYC-induced gene expression changes in the absence or presence of CB-6644. KPC*^MYC-ER^* cells were treated with DMSO or 1 µM CB-6644 for 20 hours followed by ethanol or 200 nM 4-hydroxytamoxifen (OHT) for 4 hours. Changes in total RNA (log2FC) are shown (n=3). The slope (m) and p value (p) of the linear regression (blue) are indicated as is the Pearson’s correlation coefficient (r). A line with slope m=1 is shown in black. (D) Heatmap of SLAM-seq data from cells treated as in panel C. Biological replicates are labelled 1, 2 and 3. Changes in total RNA (log2FC) are shown. (E) Sucrose gradient ultracentrifugation demonstrating a physical interaction between MYC and RUVBL1/2. Purified recombinant His6-MBP-MYC^1-163^ (MYC^1-163^, top panel), RUVBL1/2 (middle panel) and all three proteins together (bottom panel) were subjected to sucrose gradient ultracentrifugation. Fractions were collected and analysed by SDS-PAGE, followed by Coomassie blue staining. AID, auxin-inducible degron. See also online supplemental figure S5.

Next, we wanted to find out whether RUVBL1 is a critical cofactor of MYC or whether both proteins bind and regulate similar genes independently. To this end, we first analysed whether RUVBL1 chromatin binding depends on MYC. We therefore aimed to rapidly deplete MYC by the auxin-degron technology but were unable to introduce the AID sequence into the *MYC* locus in KPC cells. Instead, we could generate the desired transgenic clone in the human melanoma cell line A375. We therefore performed ChIP-seq experiments for RUVBL1 and MYC in the A375*^MYC-AID^* cells (online supplemental figure S5A). Auxin-induced depletion reduced MYC chromatin binding to background levels and strikingly reduced RUVBL1 binding at MYC-bound regions (figure 5A, online supplemental figure S5B). Overall, RUVBL1 binding was reduced in 82.7% of all joint MYC/RUVBL1 peaks, and the degree of reduction correlated with the strength of MYC binding (figure 5B). We validated the loss of RUVBL1 from promoters on depletion of MYC by ChIP-qPCR experiments (online supplemental figure S5C) and concluded that chromatin association of RUVBL1 depends on MYC.

To investigate if RUVBL1 is critical for MYC-mediated gene regulation, we combined exogenous overexpression of MYC with inhibition of the RUVBL1/2 complex. KPC cells were transduced to stably express MYC-ER, a fusion protein of MYC and the oestrogen receptor that can be activated by 4-hydroxytamoxifen (OHT). We treated cells with 200 nM OHT and analysed acute changes in transcript levels. OHT-mediated activation of MYC altered gene expression in a manner typical for oncogenic MYC (online supplemental figure S5D,E). Strikingly, incubation of cells with the RUVBL1/2 inhibitor CB-6644 prior to OHT addition globally attenuated gene activation and repression by MYC (figure 5C,D).

RUVBL1 and RUVBL2 physically and genetically interact with MYC in cells, as demonstrated previously by us^28 31^ and others.^52 53^ However, MYC also binds directly to the pseudokinase TRRAP,^22^ which is a part of the TIP60 complex together with RUVBL1 and RUVBL2. To determine if MYC binds to the RUVBL1/2 complex independently of TRRAP, we expressed MYC^1-163^ and RUVBL1 and RUVBL2 in *Escherichia coli* (online supplemental figure S5F) and analysed the purified proteins’ ability to form complexes by sucrose gradient ultracentrifugation. When tested separately, MYC^1-163^ peaked in fraction 2 and the RUVBL1/2 complex peaked in fraction 8 (figure 5E). When the proteins were incubated together before the assay, they peaked in fractions 8 and 11, respectively. This strong shift towards later fractions indicates a direct interaction between the recombinant proteins. A pull-down on recombinant MYC^1-163^ and coprecipitation of RUVBL1/2 confirmed a robust interaction (online supplemental figure S5G).

Next, we aimed to map the domains that mediate the interaction with MYC on RUVBL1 to generate interaction-deficient mutants of RUVBL1. Based on a recent report in Ewing sarcoma,^54^ we designed a series of putative loss-of-interaction mutants for RUVBL1 (RUVBL1^Δ94-118^, RUVBL1^Δ102-107^, RUVBL1^K108A^) and tested their interaction in co-immunoprecipitation experiments in KPC cells. Both RUVBL1 mutants lacking the loop in the central channel of the RUVBL1/2 hexamer (RUVBL1^Δ94-118^, RUVBL1^Δ102-107^) lost the ability to bind MYC (online supplemental figure S5H) and could not rescue the growth defect caused by auxin-mediated loss of endogenous RUVBL1 (online supplemental figure S5I). We concluded that MYC and the RUVBL1/2 complex bind to each other, explaining their co-occupancy on thousands of promoters and the relevance of RUVBL1 for MYC-mediated gene regulation and growth of pancreatic cancer cells.

### RUVBL1 is required for the maintenance and progression of pancreatic cancer

**Figure 6:**
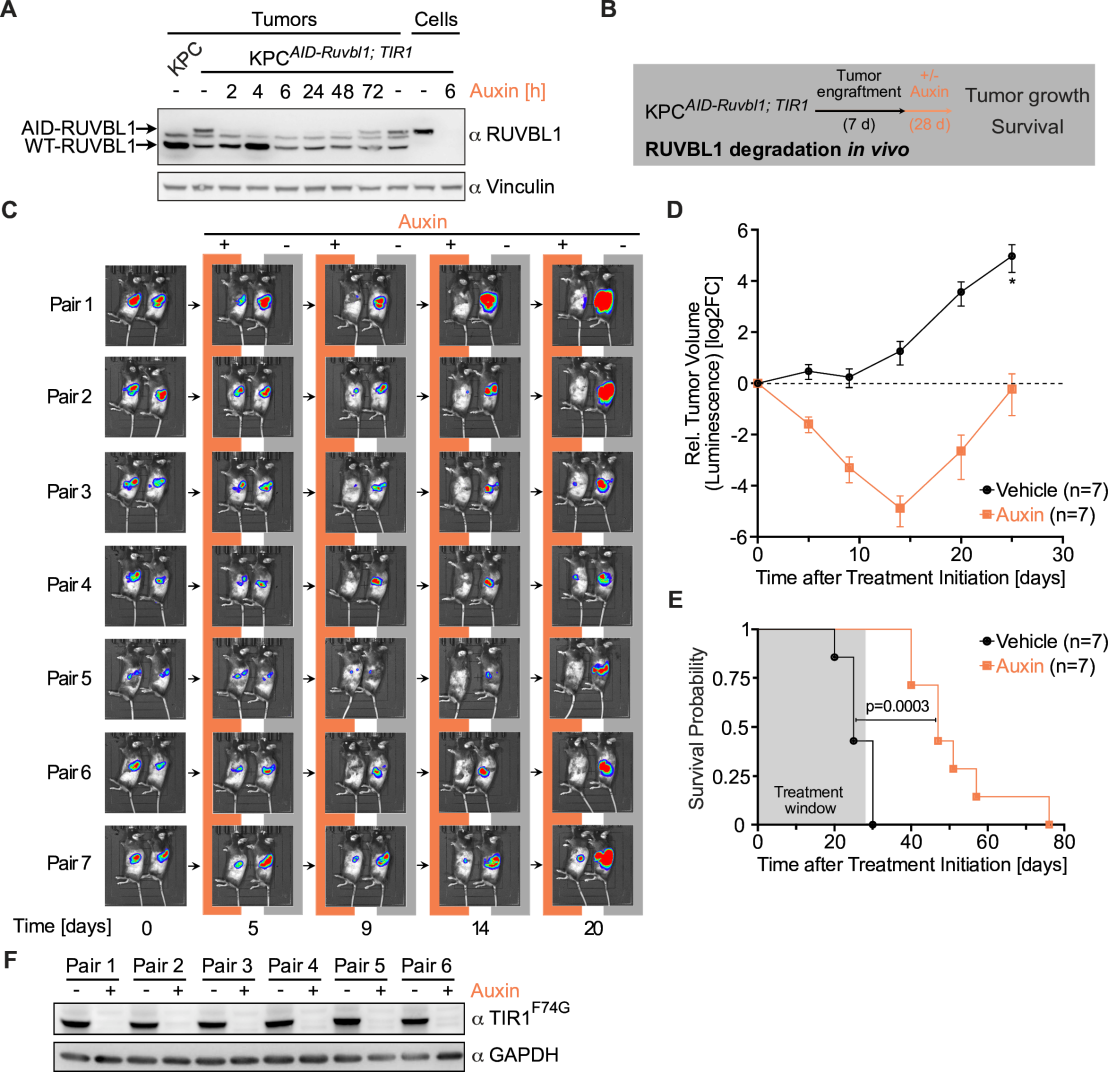
RUVBL1 is required for the maintenance and progression of pancreatic cancer. (A) Immunoblot of pancreatic tumour lysates. Native KPC cells and KPC*^AID-Ruvbl1; TIR1^* cells were transplanted into one and seven mice, respectively. After 18 days, mice with KPC*^AID-Ruvbl1; TIR1^* cell allografts were treated with 20 mg/kg auxin for up to 72 hours or with vehicle for 72 hours. Lysates of tumours were analysed using an anti-RUVBL1 antibody. Lysates of untreated and auxin-treated cultured KPC*^AID-Ruvbl1; TIR1^* cells were loaded for comparison. The band between WT and AID-tagged RUVBL1 can be attributed to murine immunoglobulin G (IgG) in the tissue. Vinculin, loading control. (B) Schematic of the in vivo RUVBL1 depletion experiment. (C) Bioluminescence images of mice with pancreatic tumours. Luciferase-expressing KPC*^AID-Ruvbl1; TIR1^* cells were transplanted into mice, and tumour size on day 7 was used to form pairs of animals with similar size tumours. Then, mice were treated daily with 20 mg/kg auxin (n=7) or vehicle (n=7). Tumour growth was assessed by bioluminescence imaging at the indicated time points. (D) Line plot of the relative volume of murine pancreatic tumours, estimated from bioluminescence as in panel C. Vehicle, n=7. Auxin, n=7. Values are mean±SEM. *n=6. (E) Kaplan-Meier survival curves for mice with pancreatic tumours treated with auxin (n=7) or vehicle (n=7). P value, Log-rank test. (F) Immunoblot of TIR1^F74G^ in lysates of tumours excised from mice that reached the humane endpoint. After KPC*^AID-Ruvbl1; TIR1^* cell engraftment, mice were treated daily with 20 mg/kg auxin or vehicle for up to 28 days. TIR1^F74G^ was detected with an antibody against the MYC tag. GAPDH, loading control. See also online supplemental figure S6.

Next, we used the AID system to acutely deplete RUVBL1 in vivo to analyse the effects of RUVBL1 on pancreatic tumour growth. To test whether auxin can reach pancreatic tumours and induce the depletion of endogenous target proteins in vivo, we transplanted KPC*^AID-Ruvbl1; TIR1^* cells into C57BL/6J mice pancreata, let tumours grow, administered various doses of auxin and isolated tumours after 6 hours. Immunoblotting showed that auxin induced the depletion of AID-tagged RUVBL1 but not of the wild-type protein expressed by stromal cells (online supplemental figure S6A). An auxin dose of 1 mg/kg body weight was effective, and the highest tested dose (20 mg/kg) did not cause visible signs of toxicity. We then examined RUVBL1 levels in tumours over time after a single auxin injection. A substantial decrease in AID-tagged RUVBL1 was seen at the earliest time point (2 hours), and the protein stayed undetectable for 24 hours (figure 6A). We assumed that daily administration of auxin would result in durable RUVBL1 depletion, enabling us to investigate the role of RUVBL1 in pancreatic tumour progression.

We next transplanted KPC*^AID-Ruvbl1; TIR1^* cells into 14 mice. Tumour size on day 7, estimated by bioluminescence imaging, was used to group mice into pairs with similar size tumours; one animal of each pair was treated daily with auxin and the other with vehicle for 28 days (figure 6B). Auxin treatment resulted in decreased bioluminescence already on day 5 and progressively lower bioluminescence until day 14, indicating that tumours were regressing drastically (figure 6C,D). However, at later time points the bioluminescence increased, and tumours returned to their starting sizes despite auxin treatment. Instead, tumours in all vehicle-treated animals grew progressively from the start of the experiment. Survival analysis showed that auxin-induced RUVBL1 depletion provided a strong survival advantage, with a median survival time of 47 vs 25 days in the vehicle-treated group (figure 6E). To understand why tumours in auxin-treated animals restarted to grow after 2 weeks, we isolated tumours when mice reached the endpoint and analysed TIR1^F74G^ levels by immunoblotting (figure 6F). Strikingly, the expression of TIR1^F74G^ was drastically reduced in all auxin-treated tumours, indicating a strong selection for cells without a functional AID system in vivo. We concluded that RUVBL1 is required for the maintenance and progression of pancreatic cancer in mice.

Since pancreatic tumours are usually detected in patients at advanced stages, we repeated the transplantation of KPC*^AID-Ruvbl1; TIR1^* cells but let tumours engraft for 16 instead of 7 days before starting auxin treatment. While all vehicle-treated mice reached the endpoint within 14 days of treatment, all auxin-treated animals survived the treatment period and had a strong overall survival benefit (online supplemental figure S6B,C). Again, in tumours excised when mice reached the endpoint, TIR1^F74G^ expression was drastically reduced in all auxin-treated animals (online supplemental figure S6D), suggesting that even advanced tumours strictly depend on RUVBL1.

### RUVBL1 promotes immune evasion in PDAC

**Figure 7:**
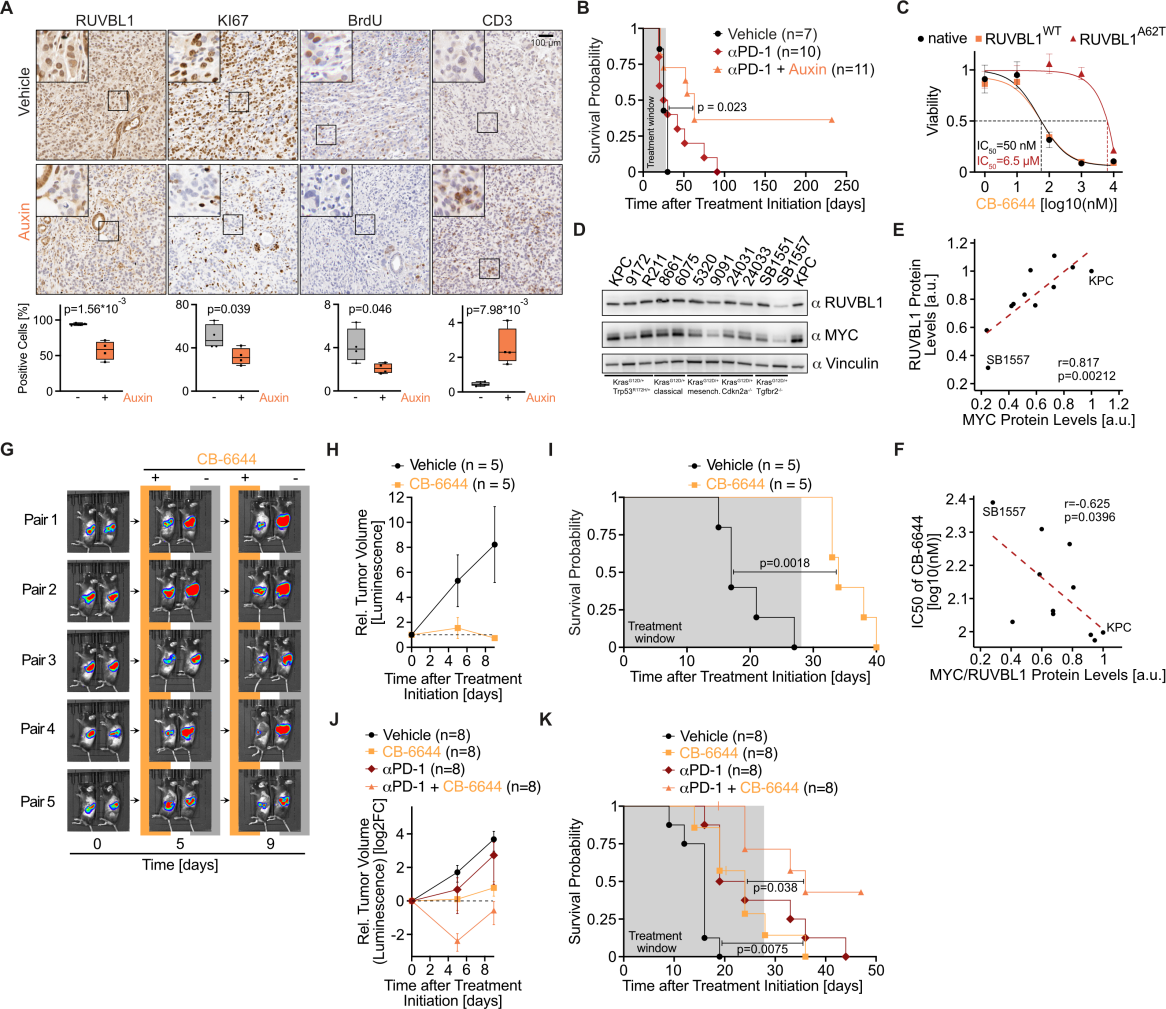
RUVBL1 promotes immune evasion in pancreatic ductal adenocarcinoma (PDAC). (A) Immunohistochemical staining of RUVBL1, KI67, BrdU and CD3 in sections of KPC*^AID-Ruvbl1; TIR1^* tumours from mice treated with vehicle (n=4) or 20 mg/kg auxin (n=4) for 5 days. Mice received an injection of BrdU and 2.5 hours later were killed to harvest the tumours. Quantification of positively stained cells is depicted below (n=4, unpaired t-test). (B) Kaplan-Meier survival curves of mice bearing KPC*^AID-Ruvbl1; TIR1^* tumours treated with 10 mg/kg anti-PD-1 antibody (αPD-1) two times per week, alone (n=10) or with 20 mg/kg auxin daily (n=11). Vehicle-treated group (n=7) from figure 6E. (C) Dose–response curves of CB-6644 on cell viability (resazurin assay) in native KPC cells and KPC cells overexpressing RUVBL1^WT^ or RUVBL1^A62T^. Cells were treated with CB-6644 for 72 hours (n=3, mean±SD). (D) Immunoblot of MYC and RUVBL1 in a panel of murine PDAC cell lines with the indicated genetic mutations representing different PDAC subtypes. (E) Scatter plot of MYC and RUVBL1 protein levels as in panel D (n=2; r, Pearson’s correlation coefficient; p value, unpaired t-test). (F) Scatter plot of MYC/RUVBL1 protein levels as in panel D and sensitivity to treatment with CB-6644 for 72 hours (n=2; r, Pearson’s correlation coefficient; p value, unpaired t-test). (G) Bioluminescence images of mice with pancreatic tumours. Luciferase-expressing KPC*^AID-Ruvbl1; TIR1^* cells were transplanted into mice, and tumour size on day 7 was used to form pairs of animals with similar size tumours. Then, mice were treated two times per day with 25 mg/kg CB-6644 (n=5) or vehicle (n=5). Tumour growth was assessed by bioluminescence imaging at the indicated times. (H) Line plot of the relative volume of pancreatic tumours in mice treated with CB-6644 (n=5) or vehicle (n=5), as in panel G. Values are mean±SEM. (I) Kaplan-Meier survival curves for mice with pancreatic tumours treated with CB-6644 (n=5) or vehicle (n=5), as in panel G. Log-rank test. (J) Line plot of the relative volume (mean±SEM) of pancreatic tumours in mice treated with CB-6644 (n=8), αPD-1 (n=8), a combination of αPD-1 and CB-6644 (n=8) or vehicle (n=8). KPC cells were transplanted into mice, and tumour size on day 7 was used to form pairs of animals with similar size tumours. Mice were treated two times per day with 25 mg/kg CB-6644, two times per week with 10 mg/kg αPD-1, a combination of both or vehicle for up to 28 days. (K) Kaplan-Meier survival curves for mice with pancreatic tumours treated with CB-6644 (n=8), αPD-1 (n=8) or a combination of both (n=8), as in panel J. Log-rank test. See also online supplemental figure S7.

We found it intriguing that RUVBL1 depletion triggered strong tumour regression in vivo, whereas the same tumour cells only arrested in culture. We therefore examined tumours histologically after 5 days of auxin treatment to study the underlying cellular mechanisms. Immunohistochemistry confirmed the auxin-mediated depletion of RUVBL1 in tumour cells (figure 7A), while RUVBL1 levels in the surrounding healthy tissue and stromal cells were not affected (online supplemental figure S7A). In addition, we observed a loss of the proliferation marker KI67 and a sharp decrease in BrdU incorporation in auxin-treated tumours, consistent with the phenotype of reduced growth of cultured KPC cells. Since MYC promotes immune evasion in pancreatic tumours,^12 35 51^ we analysed immune cell infiltration on RUVBL1 depletion and observed a rampant increase in CD3-positive cells within tumours on auxin treatment (figure 7A, online supplemental figure S7A). We concluded that RUVBL1 depletion in pancreatic tumours induces immune cell infiltration and therefore phenocopies the genetic silencing of MYC in similar tumour models.

Human pancreatic tumours usually contain few immune cells, which is thought to explain their low response to immune checkpoint blockade. We therefore combined auxin-mediated depletion of RUVBL1 with administration of an αPD-1 antibody. Strikingly, combinatorial treatment, but not αPD-1 treatment alone, induced long-term survival of four of the 11 mice even after the treatment was terminated (figure 7B). We concluded that depletion of RUVBL1 in pancreatic cancer leads to immune infiltration and induces tumour sensitivity to immune checkpoint blockade.

### Oncogenic expression of MYC renders cells dependent on RUVBL1

We wondered whether the dependence of pancreatic tumours on the RUVBL1/2 complex is conveyed by high MYC expression, so we did a series of experiments using the RUVBL1/2 inhibitor CB-6644 to address this question. To first investigate whether the toxicity of CB-6644 in KPC cells is due to specific inhibition of RUVBL1, we stably expressed an inhibitor-resistant RUVBL1 mutant (RUVBL1^A62T^).^49^ Both native KPC cells and KPC cells overexpressing wild-type RUVBL1 were sensitive to CB-6644 (IC_50_=50 nM). Expression of RUVBL1^A62T^ but not the catalytically inactive double-mutant RUVBL1^A62T, D302N^ reduced the cells’ sensitivity by more than 100-fold (IC_50_=6.5 µM, figure 7C, online supplemental figure S7B), indicating that CB-6644-induced toxicity is mediated by the catalytic inhibition of RUVBL1.

We then analysed whether MYC expression is responsible for the sensitivity of pancreatic cancer cells to RUVBL1 inhibition. To this end, we first quantified MYC and RUVBL1 levels in a panel of pancreatic cancer cells by immunoblotting (figure 7D) and observed a strong correlation between the levels of both proteins (figure 7E) and between their levels and sensitivity to CB-6644 as estimated in cell growth assays (figure 7F).

Subsequently, we silenced MYC in KPC cells with a doxycycline-inducible shRNA (online supplemental figure S7C) and treated them with 200 nM CB-6644. In cells not exposed to doxycycline, viability was strongly reduced (89±14%) by CB-6644 treatment. In contrast, silencing of MYC by doxycycline treatment rendered the cells largely resistant to CB-6644 (viability reduction, 31±4%, online supplemental figure S7D). We concluded that high MYC expression causes tumour cells to depend on the complete RUVBL1 protein function.

We next investigated whether CB-6644 also exerts its antitumour effect on human cancer cells. We tested a panel of six human PDAC cell lines for their sensitivity to CB-6644 and obtained IC_50_ values between 12 and 110 nM (online supplemental figure S7E). We concluded that the dependency on RUVBL1 is not restricted to the murine KPC cell system but is also observed in human PDAC cells.

Since the complete loss of RUVBL1 is embryonically lethal,^55^ we analysed if CB-6644 affects pancreatic cancer progression in vivo at tolerated doses. We transplanted KPC cells into 10 mice, and based on tumour size on day 7, mice were grouped into pairs with similar size tumours; one animal of each pair was treated twice daily with CB-6644 and the other with vehicle. Bioluminescence imaging showed that tumours in CB-6644-treated mice stopped growing or even regressed, while tumours in vehicle-treated animals steadily progressed (figure 7G,H). This finding is remarkable, since the concentration of CB-6644 was higher in all tested healthy tissues than in pancreatic tumours (34 times higher in spleen, online supplemental figure S7F) and since the limited solubility of CB-6644 prevented the use of higher, possibly more effective doses. Strikingly, no CB-6644-treated mouse reached the endpoint during the 28-day treatment, while all vehicle-treated mice did (figure 7I). Thus, the median survival time of tumour-bearing mice was doubled by CB-6644 treatment. We next wondered if CB-6644-mediated inhibition of RUVBL1 would also synergise with αPD-1 treatment, as it did with auxin-mediated depletion. We therefore transplanted naïve KPC cells into 32 mice and, based on tumour size on day 7, sorted the mice into groups with similar size tumours, each receiving CB-6644 or αPD-1 treatment, combinatorial treatment or vehicle injections. While both CB-6644 and αPD-1 mono treatments slowed tumour growth, the combinatorial treatment induced tumour regression (figure 7J) and most profoundly extended the life span of tumour-bearing mice (figure 7K). Finally, inhibition of RUVBL1 by CB-6644 for 5 days caused infiltration of CD3-positive immune cells in tumours induced by a second KPC cell line (9172) and murine PDAC cells driven by the mutations *Kras^G12D/+^* and *Cdkn2a^−/^* ^−^ (24031), indicating that RUVBL1-mediated immune evasion is not restricted to the initially used KPC cell line (online supplemental figure S7G,H). Overall, we concluded that high MYC expression renders pancreatic cancer cells dependent on RUVBL1/2 and that inhibition of the RUVBL1/2 complex is therapeutic at doses tolerated by healthy tissues.

## Discussion

The hallmark oncogene MYC plays a crucial role in tumorigenesis and is considered a key therapeutic target based on many studies in murine tumour models.^7 10 16^ Here, we explored the approach of targeting MYC via its interactome. MYC depends on its binding partners for function, but it was unclear which of these are most relevant for oncogenic growth and tumour maintenance.

To narrow down the spectrum of MYC binding partners, we performed a series of genetic screens in cultured PDAC cells and fibroblasts, as well as in murine pancreatic tumours. Among the candidates essential for PDAC growth in vivo but dispensable for fibroblasts, the two complex-forming proteins RUVBL1 and RUVBL2 scored highest. RUVBL1 depletion mimicked cellular phenotypes previously observed on MYC depletion in pancreatic cancer cells. For instance, depletion of RUVBL1 (in this study) or MYC^26 35^ resulted in S-phase arrest but not apoptosis in cultured cells. Furthermore, depletion of either protein in immunocompetent mice led to the rapid regression of pancreatic tumours accompanied by immune cell infiltration, as shown here for RUVBL1 and earlier for MYC.^12 35 51^

Consistent with these phenotypic similarities is our observation that high MYC expression levels make cancer cells dependent on the full activity of RUVBL1. We therefore consider the MYC-RUVBL1 axis to be a targetable vulnerability in cancer cells, since the RUVBL1/2 complex is a druggable AAA ATPase.^56^ In fact, the allosteric RUVBL1/2 inhibitor CB-6644 had previously been shown to arrest Ewing sarcoma and multiple myeloma in immunocompromised mice^49 54^ and had a striking therapeutic effect on pancreatic tumours at tolerated concentrations in immunocompetent mice in our study. However, because of the drug’s low solubility and unfavourable pharmacokinetics, it was not possible to establish higher, possibly even more effective drug concentrations in pancreatic tumours. Nonetheless, we expect that it will be possible to develop inhibitors of RUVBL1 with a better pharmacokinetic profile.

In addition to the important identification of RUVBL1 as a candidate for the development of an MYC-based cancer therapy, several further observations emerged from this study. First, we found that the dependence of pancreatic cancer cells on specific MYC binding partners differs dramatically when grown in culture or in vivo. About one-third of all tested MYC binding partners were essential for KPC cells in culture but dispensable for the same cells when growing in their natural environment in mice. Some of these proteins are currently targets of drug development campaigns, such as YY1, p400 and RAD21. We speculate that unnatural metabolite conditions and the absence of stromal cells in culture generate artificial dependencies. On the other hand, the settings of our in vivo screen could also create artificial dependencies, such as the immunogenicity of transgenes and the use of doxycycline for shRNA activation. Overall, our results highlight the need to complement the available results of genome-wide screens in cancer cell line panels^36^ with genetic screens in intact organisms. However, our in vivo screen did not only devalidate target candidates but also confirmed proteins as promising cancer targets. One prominent example is WDR5, which was one of the few tested MYC binding partners with an even greater dropout in pancreatic tumours than in cultured KPC cells. WDR5 is considered a druggable cancer target and is being intensively investigated by others.^57–59^

Our work also clarified the mechanistic connection between MYC and RUVBL1. It has been shown earlier that MYC recruits the TIP60 complex to chromatin and interacts with the TIP60 components TRRAP and RUVBL1/2^27 52 53^ via a domain known as MYC Box II, which is essential for MYC’s transforming potential.^29^ The role of RUVBL1 on MYC-mediated gene regulation was however unclear. To understand the primary effect of RUVBL1 on gene expression, we used the AID system to induce the complete degradation of RUVBL1 within a few hours. The acute depletion of RUVBL1 induced the downregulation of a gene expression profile typical of high, oncogenic MYC expression. In addition, promoters of RUVBL1-activated genes were bound by both MYC and RUVBL1, suggesting direct activation of RNAPII function (see graphical abstract). In contrast, we hypothesise that the regulation of MYC/RUVBL1 repressed genes is indirect, since the promoters of these genes are much less occupied by both proteins. We speculate that in a RUVBL1-proficient situation, RNAPII or RNAPII-associated proteins are recruited to MYC/RUVBL1-bound genes and thus sequestered away from MYC/RUVBL1-negative promoters. Such a squelching mechanism has previously been proposed by us^24^ and others^60^ for MYC-mediated gene repression.

This study also demonstrated that the AID system can be used to degrade RUVBL1 in vivo. Previously, this system was shown to degrade GFP reporter proteins in healthy mouse tissues,^48^ but it was unclear whether it could also be used for target depletion in orthotopic tumours in immune-competent mice. The established auxin depletion system can be used to investigate whether other murine and human PDAC models are similarly dependent on RUVBL1 as the KPC cell model used here. This is necessary because human PDAC tumours are characterised by a high degree of phenotypic and genetic heterogeneity and may therefore show variable dependence on RUVBL1. The auxin system is also ideal for investigating whether the effects of RUVBL1 on transcription are always MYC dependent, since RUVBL2 has been shown to have direct effects on the subnuclear distribution and clustering of RNAPII in mouse embryonic stem cells.^47^ Overall, we envision that auxin-mediated target depletion, in combination with bioluminescence, will become a powerful tool for future mechanistic and translational studies in mice.

Finally, we observed that tumour regression after RUVBL1 depletion was accompanied by a massive infiltration of CD3-positive immune cells in the KPC model used here. This finding is significant, since both murine and human pancreatic tumours are sparse in immune cells and are therefore considered immunologically ‘cold’.^61^ Accordingly, most patients with pancreatic cancer do not benefit from immunotherapies such as PD-1/CTLA-4 checkpoint blockade. We speculate that drugs targeting the MYC-RUVBL1 axis could make pancreatic tumours susceptible to immunotherapy. In support of this proposition, four of 11 mice with orthotopic tumours were cured by a combinatorial treatment consisting of transient RUVBL1 depletion and PD-1 checkpoint blockade. Thus, this study has possibly discovered a pharmaceutical strategy to render pancreatic cancers immunologically ‘hot’ and susceptible to immune therapy.

## Methods

### Cell lines and animals

KPC (kindly provided by Jens Siveke, Essen, Germany), 9172, R211 (Kras^G12D/+^, Trp53^R172H/+^), 8661, 6075 (Kras^G12D/+^, classical), 5320, 9091 (Kras^G12D/+^, mesenchymal), 24031, 24033 (Kras^G12D/+^, Cdkn2a^−/−^), SB1551, SB1557 (Kras^G12D/+^, Cdkn2a^−/−^) (kindly provided by Dieter Saur, Munich, Germany), NIH3T3 (RRID:CVCL_0594), A375 (RRID:CVCL_0132), PaTu 8988t (RRID:CVCL_1847) and PANC-1 (RRID:CVCL_0480) cell lines were cultured in DMEM (Thermo Fisher Scientific) supplemented with 10% FBS (Capricorn Scientific), 100 U/mL penicillin and 100 µg/mL streptomycin (Sigma-Aldrich) at 37°C, 5% CO_2_. IMIM-PC1 (RRID:CVCL_4061), AsPC1 (RRID:CVCL_0152), BxPC-3 (RRID:CVCL_0186) and Panc 08.13 (RRID:CVCL_1638) cells were cultured in RPMI-1640 medium supplemented with 10% FBS, 100 U/mL penicillin, 100 µg/mL streptomycin (Sigma-Aldrich) and 1× MEM Non-Essential Amino Acids Solution (Thermo Fisher Scientific) at 37°C, 5% CO_2_. Cell lines were routinely subjected to PCR-based *Mycoplasma* testing and at all times tested negative.

C57BL/6J mice originating from the Jackson Labs (RRID:IMSR_JAX:000664) were used for producing the inbred colony. Mice were housed in pathogen-free conditions on a 12-hour dark/light cycle with unlimited access to food and water. All experiments were performed with males aged 8–12 weeks. The ARRIVE reporting guidelines for animal research (Animal Research: Reporting of In Vivo Experiments) were applied.^62^

### Consensus set of MYC binding partners and essentiality scores

The proteins to be targeted by the shRNA library were selected from our MYC interactome analyses published in Baluapuri *et al*.^28^ Well-accepted MYC binding partners were added to this list, and MYC was included as a positive control. A list of all MYC interactors included in the library can be found in online supplemental table 1.

For each chosen MYC binding partner, we determined if it was annotated as a ‘common essential gene’ according to CRISPR screens in the DepMap portal (depmap.org; 21Q3 data release). Furthermore, we calculated the percentage of cancer cell lines in the DepMap database for which the gene was considered ‘common essential’. We termed this percentage the ‘essentiality score’. We considered genes with an essentiality score equal to or greater than 50% as ‘common essential’.

### shRNA library cloning; sequencing and assessment of representation

For each chosen MYC binding partner and MYC itself, five 97-mer shRNA oligonucleotides were designed using SplashRNA, a sequential classification algorithm.^63^ For NTCs, we designed 18 shRNA sequences against green fluorescent protein or *Renilla* luciferase. Oligonucleotides were purified on a reverse phase cartridge (Sigma-Aldrich), pooled and PCR amplified using the primers mirE_XhoI_f and mirE_EcoRI_r (online supplemental table 6). The PCR products were digested with EcoRI/XhoI (New England Biolabs) and ligated into the inducible miR-E expression vector LT3GEPIR (Addgene: 111177) using T4 ligase (Thermo Fisher Scientific).

10.1136/gutjnl-2023-331519.supp7 Supplementary data

The resulting library was assessed by next-generation sequencing. First, 2 ng plasmid DNA was PCR amplified using primers containing Illumina adapters (mirE_NGS_PCR1_f/r) (online supplemental table 6) as follows: template DNA was initially denatured at 98°C for 2 min and then amplified over 18 cycles consisting of denaturation (10 s at 98°C), primer annealing (20 s at 65°C) and extension (30 s at 72°C). A final extension was done for 5 min at 72°C. PCR products were purified using the GeneJET Gel Extraction kit (Thermo Fisher Scientific) and amplified a second time with Illumina-compatible dual barcoded primers (i7/i5 index primer, online supplemental table 6). These PCR conditions included an initial denaturation step (5 min at 98°C), followed by nine cycles of denaturation (10 s at 98°C), primer annealing (20 s at 65°C) and extension (30 s at 72°C), followed by 5 min at 72°C for a final extension.

The PCR-amplified shRNA library was sequenced on an Illumina NextSeq500 sequencer. FASTQ files were aligned to the shRNA reference sequences using Bowtie sequence aligner (version 1.2.2) allowing no mismatch (-v 0). Alignments per shRNA sequence were counted using SAMtools version 1.7, and differential enrichment was analysed using DESeq2 version 1.36.0. The distribution of the shRNA library was assessed by plotting the kernel density estimate using ggplot2 (version 3.3.6). Uniform representation of the library was defined as: (1) all shRNAs identified by sequencing, and (2) abundance differing by less than 10-fold for >80% of all shRNA sequences.

### shRNA genetic screens

The shRNA library in the LT3GEPIR backbone was transfected together with lentiviral packaging plasmids psPAX2 (Addgene: 12260) and pMD2.G (Addgene: 12259) into HEK293T cells using polyethylenimine (Sigma-Aldrich). The lentiviral supernatant was harvested 24 and 48 hours after transfection, pooled and used immediately for infections.

For the in vitro cell culture screen, murine KPC pancreatic cancer cells were infected in triplicate with lentivirus at a multiplicity of infection of 0.1 for 48 hours. Then, cells were selected with 2 µg/mL puromycin (InvivoGen) for 72 hours. Afterwards, each transduced KPC culture was divided into two: in one-half the culture medium was supplemented with 1 µg/mL doxycycline (Sigma-Aldrich) and the other half received an equal volume of ethanol vehicle. The six cultures were subcultured every 2–3 days while maintaining a representation of at least 1000 cells per shRNA construct. Cells were harvested after 14 days of treatment, and the pellets were snap frozen before analysis. The same procedure was used for transduction of NIH3T3 fibroblasts.

For the in vivo screen, KPC cells transduced with the shRNA library were further transduced to express firefly luciferase. For this, firefly luciferase in the pRRLSin.cPPT.SFFV-IRES-Hygro.WPRE backbone (pRRLSin.cPPT.PGK-GFP.WPR (Addgene: 12252), where PGK was exchanged with the SFFV promoter) was used for the production of lentiviral supernatant as described above. KPC cells were infected with lentivirus for 48 hours and then selected with 500 µg/mL hygromycin (InvivoGen) for 7 days.

KPC cells transduced with the shRNA library and the firefly luciferase vector were orthotopically injected into pancreata of C57BL/6J mice as described in the ‘Pancreatic allografts’ section. First, we determined the best number of cells to inject into pancreata to form tumours with uniform representation of the library (without doxycycline induction) by injecting 50 000 cells and 100 000 cells into one mouse each. The mice were fed standard chow (ssniff Spezialdiäten) ad libitum, and when mice reached the humane endpoint, they were killed and the tumours were harvested. Reaching the humane endpoint was determined from both signs of reduced well-being (dishevelled fur, blurry eyes, reduced interactions, lethargy) and large tumour size (assessed by bioimaging). Genomic DNA was extracted from tumours and assessed for shRNA representation. The best cell number was then injected into the pancreata of 10 C57BL/6J mice. After 7 days, mice were divided into five pairs based on the size of developed tumours. One mouse of each pair was fed chow containing 625 mg/kg doxycycline (A112D70624, ssniff Spezialdiäten), while the other mouse received standard chow (both, ad libitum). After 14 days, the tumours were explanted and snap frozen.

Cultured cells and tumour-derived cells from the screens were lysed in 10 mM Tris-HCl pH 8, 100 mM NaCl, 10 mM EDTA, 0.5% SDS, 400 µg/mL proteinase K at 56°C overnight. Lysates were treated with 100 µg/mL RNAse A (7156.1, Carl Roth) for 1 hour at 37°C. Genomic DNA was sheared by sonication and purified by phenol/chloroform/isoamyl alcohol (Carl Roth) extraction and isopropanol precipitation. Then, 3 µg DNA was used to prepare libraries for sequencing as described above for the shRNA plasmid library (six PCR reactions, 500 ng each). The libraries were PCR amplified with 26 cycles in the first and nine cycles in the second round, and then sequenced and analysed as above.

For data analysis, the 18 NTC shRNAs were randomly binned into two groups of five and two groups of four to create groups of control shRNAs similar in size to those of the targeting sequences. Groups NTC1 and NTC2 were assigned five sequences, while groups NTC3 and NTC4 had four sequences.

To assess the quality of the in vivo screen, volcano plots were used to compare control tumours (standard chow) and the cells that were transplanted (input), as well as to compare doxycycline-treated and control tumours. The median log2FC of five shRNAs per gene (or NTC groups) was used to create waterfall plots and scatter plots comparing the screens. An essential gene was defined as log2FC <−1, and a dispensable (non-essential) gene was defined as one with log2FC >−1.

### Pancreatic allografts

For all animal experiments, the sample size per group was determined by a priori power analysis. Tumour size was the primary outcome measure. For the orthotopic transplantation of KPC cells transduced with the shRNA library, KPC*^AID-Ruvbl1; TIR1^* cells, native KPC cells, 9172 cells or 24031 cells into C57BL/6J mice pancreata, cells were suspended in 50 µL of 50% Geltrex LDEV-Free Reduced Growth Factor Basement Membrane Matrix (Thermo Fisher Scientific) in phosphate buffered saline (PBS). Mice were anaesthetised by the intraperitoneal injection of 100 mg/kg body weight ketamine (Ratiopharm) and 10 mg/kg xylazine (Bayer). A 0.5–1.0 cm incision was made through the skin below the ribcage on the left side of the abdomen and above the spleen. The peritoneum was cut at the same location. The spleen was gently lifted with a cotton swab so that the pancreas became accessible. The cells were injected subcapsularly into the pancreas, causing the formation of a fluid bubble. The pancreas was then placed back into the abdomen. The peritoneum was closed with two to five single-head sutures, and the skin wound was closed with 7 mm clips.

An IVIS Lumina Series III in vivo imaging system (Perkin Elmer) was used to measure the bioluminescent signal generated by tumour cells expressing firefly luciferase as a proxy for tumour growth. For this purpose, 150 mg/kg luciferin D (Biozol, Eching, Germany) was intraperitoneally administered. After 10 min, mice were anaesthetised by inhalation of 1.5–3% isoflurane (CP Pharma) in O_2_ for the duration of the measurement. Imaging was performed 7 days after tumour induction and then every 4–5 days for the duration of experiments.

Animals were allocated to a treatment group based on bioluminescent imaging before the start of the treatment. For the anti-PD-1/auxin combinatorial treatment, animals with similar tumour sizes (measured by luminescence) after 7 days were used. For all other experiments no animals were excluded. Two mice with the most similar luminescence signals were paired. In each pair, one randomly selected mouse received the experimental treatment (ie, auxin, CB-6644 or combined treatment of auxin and anti-PD-1 antibody) and the other mouse the comparator treatment (vehicle or only anti-PD-1 antibody), depending on the experiment. Pairs were treated and imaged at the same time to minimise potential confounders. Experimenters were not blinded for the group allocation.

In experiments with KPC*^AID-Ruvbl1; TIR1^* allografts, mice were treated intraperitoneally with 1–20 mg/kg auxin (5-phenyl-1H-indole-3-acetic acid; BioAcademia) in 10% DMSO, 90% PBS or with vehicle (10% DMSO, 90% PBS) daily. In the experiment where synergism between genetic degradation of RUVBL1 and blockage of PD-1 signalling was assessed, in addition to the auxin treatment as already described, mice were intraperitoneally injected with 10 mg/kg anti-PD-1 blocking antibody (Biozol) two times per week. The RUVBL1/2 inhibitor CB-6644 (MedChemExpress)^49^ was administered two times per day at 25 mg/kg in 10% DMSO, 90% PBS. BrdU (BD Pharmingen) was injected intraperitoneally at 50 mg/kg 2.5 hours prior to sacrificing. Toxicity of treatments was assessed by monitoring the animals’ well-being and by scoring their body condition.

Late-stage tumours were those allowed to grow for 16 days before auxin treatment was started. All in vivo experiments were terminated before or when mice reached the humane endpoint, as described above.

Differences in survival were tested using the log-rank test and p values were calculated using GraphPad PRISM.

### Analysis of public expression data

TCGA mRNA expression data and associated clinical data were downloaded from https://portal.gdc.cancer.gov/. The curated data set was used for all analysis.^45^ The Hallmark MYC Target V1 gene expression score was defined as the mean expressed gene of the HALLMARK_MYC_TARGET_V1 gene set per patient after scaling the expression of every individual gene across all patients in the TCGA database. Scores for basal-like PDAC (basal-like subtype, pancreatic cancer subtypes, WP5390) and undifferentiated cancer (RHODES_UNDIFFERENTIATED_CANCER, C2, MSigDB) were calculated in the same way. For survival analysis, patients with PDAC were stratified into groups of high and low RUVBL1 and MYC target gene expression (top and bottom third) and survival of the patients with the 50% most aggressive diseases was plotted. The p value was calculated using log-rank test. The overexpression of MYC binding partners in PDAC compared with healthy pancreas was assessed by downloading raw RNA-seq count tables for all patients with PDAC (178 primary tumour samples, 4 healthy pancreas samples) and conducting differential gene expression analysis using the DESeq2 pipeline. Expression and survival analyses were repeated with the ICGC PACA-CA data set containing Canadian patients with PDAC.

### Immunoblotting

Cells were lysed in RIPA lysis buffer (50 mM HEPES pH 7.9, 140 mM NaCl, 1 mM EDTA, 1% Triton X-100, 0.1% SDS, 0.1% sodium deoxycholate) supplemented with phosphatase and protease inhibitors (phosphatase inhibitor cocktail 2/3, P5726, P0044; protease inhibitor cocktail, P8340, Sigma-Aldrich) at 4°C head over tail for 20 min. Lysates were cleared by centrifugation, and the supernatant was collected. Protein was quantified using the BCA assay, and samples were separated using Bis-Tris-PAGE. Vinculin (1:5000; V9131, Sigma-Aldrich) and GAPDH (1:1000; 2118, Cell Signaling) were used as loading controls.

Separated proteins were transferred to PVDF membranes (Merck Millipore) and incubated with 5% (w/v) non-fat dry milk in TBS-T (20 mM Tris-HCl pH 7.5, 150 mM NaCl, 0.1% (v/v) Tween-20) for 1 hour at room temperature for blocking. The membranes were washed and incubated with primary antibodies (RUVBL1, 1:1000, 74775, Cell Signaling; RUVBL2, 1:5000, sc374135X, Santa Cruz Biotechnology; MYC, ab32072, Abcam; total RNAPII, sc17798, Santa Cruz Biotechnology; AID tag, 1:1000, MBL-M214-3, MBL International; MYC tag, 1:500, 05-419, Sigma-Aldrich) overnight at 4°C. Bands were visualised using a horseradish peroxidase (HRP)-conjugated secondary antibody (anti-rabbit immunoglobulin G (IgG), 1:7500, 1079-4347/GEHENA934, GE Healthcare; anti-mouse IgG, 1:7500, 1019-6124/GEHENA931-1ML, GE Healthcare) and Immobilon Western HRP substrate (WBKLS0500, Merck Millipore). Blot images were acquired using a LAS 4000 Mini Gel Imager (Fuji) and quantified using Image Studio Lite quantification software (V.5.2.5; LI-COR Biosciences).

### Cellular growth assays

Cell growth was quantified by seeding 50 000 KPC cells per well in 6-well plates and treating with auxin or DMSO for 10 days. Cells were counted every 2–3 days using a CASY cell counter and reseeded at 50 000 cells/well. Cell viability was assessed using the alamarBlue (resazurin) reagent (Thermo Fisher Scientific). 10 µL per 90 µL medium was added to the cell culture vessel. After 2 hours, fluorescence was measured in a Spark microplate reader (Tecan). Colony-forming capacity was assayed by seeding 20 000 KPC cells per well in 6-well plates and treating them for 4 days if not stated otherwise. Cells were stained with crystal violet.

### Flow cytometry

To evaluate the cell cycle profile and replication capacity of KPC*^AID-Ruvbl1; TIR1^* cells, 50 000 cells were treated with auxin or DMSO vehicle for 24, 48 and 72 hours and labelled with 10 µM BrdU (Sigma-Aldrich) for 1 hour. The cells were collected by trypsinisation together with their supernatant, centrifuged, washed twice with ice-cold PBS and fixed in 80% ethanol at −20°C overnight. Fixed cells were washed with ice-cold PBS and resuspended in 2 M HCl, 0.5% Triton X-100 for 30 min at room temperature. 0.1 M Na_2_B_4_O_7_ (pH 8.5) was added for neutralisation. Pellets were resuspended in 100 µL PBS-T (0.5% Tween-20 in PBS) containing 1% BSA and 1 µg FITC-labelled anti-BrdU antibody (BioLegend). After a 30 min incubation at room temperature in the dark, cells were washed with 1% BSA in PBS-T and resuspended in PBS containing 24 µg/mL RNAse A (Carl Roth) and 54 µM propidium iodide (Sigma-Aldrich). After incubation at room temperature for 30 min in the dark, cells were analysed on a BD FACSCanto II flow cytometer. Data were analysed with BD FACSDIVA (V.6.1.2) and FlowJo (V.8.8.6) software.

### Endogenous knock-in of aid tag and targeted degradation

The CRISPR knock-in of the AID sequence^64^ into the murine *Ruvbl1* locus was performed by cloning a homology-directed repair (HDR) template containing homology arm (HA) homologues to the sequences upstream and downstream of the *Ruvbl1* start codon. HAs were PCR amplified and cloned into pJET (Thermo Fisher Scientific) flanking the Blast-P2A-FLAG-AID cassette. An sgRNA targeting the region around the *Ruvbl1* start codon was cloned into PX458 (Addgene: 48138). sgRNA plasmid and HDR template were cotransfected into KPC cells with Lipofectamine 2000 (Thermo Fisher Scientific). After 72 hours, cells were selected with 15 µg/mL blasticidin (InvivoGen) for 10 days. Colonies from single-cell clones were transferred to 24-well plates and genotyped by PCR. PCR products were purified using the GeneJET Gel Extraction kit (Thermo Fisher Scientific) and sent for Sanger sequencing (LGC Genomics). The used KPC*^AID-RUVBL1^* clone (C6) is a hemizygous clone in which the AID tag was successfully integrated into one *Ruvbl1* allele, while in the second allele deletion of the start codon abrogated expression (see online supplemental figure 3B,C).

CRISPR knock-in of the AID sequence was also done at the MYC locus of human cells as described above, with the following modifications: HAs flanked the MYC stop codon and, after PCR, were cloned into pJET flanking the AID-V5-P2A-Blast cassette. The sgRNA targeted the region around the MYC stop codon, and this plasmid and the HDR template were cotransfected into A375 human melanoma cells.

For TIR1^F74G^ expression in KPC*^AID-Ruvbl1^* cells, TIR1^F74G^ was cloned into pRRLSin.cPPT.SFFV-IRES-Hygro.WPRE. KPC*^AID-Ruvbl1^* cells were lentivirally transduced with the vector and selected with 500 µg/mL hygromycin (InvivoGen) for 7 days. AID-tagged protein degradation was induced by treatment with 1 µM auxin (30-003-10, BioAcademia), if not stated otherwise. The auxin vehicle, DMSO, was used in experiments as the negative control. Firefly luciferase was cloned into the pRRLSin.cPPT.SFFV-IRES-Puro.WPRE backbone (we used pRRLSin.cPPT.SFFV-IRES-Hygro.WPRE with the hygromycin resistance sequence exchanged to puromycin). KPC*^AID-Ruvbl1; TIR1^* cells were infected with lentiviral supernatant and selected with 2 µg/mL puromycin (InvivoGen) for 72 hours.

### SLAM-seq

Cells were treated with auxin for 3 or 15 hours and subsequently with 800 µM 4sU (Sigma-Aldrich) for 2 hours. Alternatively, they were treated with 1 µM CB-6644 (MedChemExpress) for 20 hours, followed by 200 nM OHT (H7904, Sigma-Aldrich) for 4 hours and 400 µM 4sU for 2 hours. Cells were harvested in QIAzol lysis reagent (Qiagen), and RNA was extracted by phenol/chloroform/isoamyl alcohol (Carl Roth) extraction and precipitation with isopropanol (Carl Roth). Incorporated 4sU was alkylated using 10 mM iodoacetamide (Thermo Fisher Scientific), and the reaction was quenched with 1 M DTT (Thermo Fisher Scientific). Alkylated RNA was purified on MinElute columns (Qiagen). RNA integrity was verified using the Standard Sensitivity RNA kit (Agilent Technologies) on a Fragment Analyzer system (Agilent Technologies). Samples that passed quality checks were used for library preparation using the QuantSeq Fwd kit (Lexogen) for 15 cycles. They were sequenced for 75 cycles on a NextSeq500 or for 120 cycles on a NextSeq2000 sequencer (Illumina).

We used the GRAND-SLAM pipeline (V.2.0.7) to process both SLAM-seq data sets. Briefly, 10 nt (6 nt unique molecular identifier (UMI)+4 nt spacer) were trimmed from the 5' ends of reads (FastqFilter program from the GRAND-SLAM pipeline), and the sequencing adapter (AGATCGGAAGAGCACACGTCTGAACTCCAGTCA) was trimmed from the 3' end using Cutadapt V.3.4. Next, Bowtie 2 (V.2.3.0) with default parameters was used to discard reads mapping to rRNA (GenBank identifier U13369.1) and to verify the absence of *Mycoplasma* contamination. STAR V.2.5.3a was used to map all remaining reads with a length of at least 18 nt against a combined index of the murine genome (Ensembl 102) and ERCC92 spike-ins (parameters: --outFilterMismatchNmax 20, --outFilterScoreMinOverLread 0.4, --outFilterMatchNminOverLread 0.4, --alignEndsType Extend5pOfReads12, --outSAMattributes nM MD NH). Finally, all reads mapping to the same genomic location sharing the same UMI were collapsed, and only mismatches that occurred in the majority of these reads were retained (DedupUMI program of the GRAND-SLAM pipeline). The GRAND-SLAM program was run with parameter –trim 15 against a combined index of murine mRNAs (Ensembl 102) and the ERCC92 spike-ins, to count reads and to estimate the new-to-total RNA ratio for each sample and each mRNA.

The grandR package (version 0.1.11 for the CB-6644 data set, and version 0.1.23 for the auxin data set) was used for quality control and downstream analyses. The absence of cellular toxicity of 4sU was confirmed using the ‘PlotToxicityTestRankAll’ function. Genes were filtered to have at least 50 reads in at least half of the samples. Read counts per sample were normalised by dividing by the size factors (estimateSizeFactorsForMatrix from the DESeq2 R package) computed from either the total number of ERCC92 mapped reads or the total number of murine mRNA mapped reads. Greater within-replicate variability after ERCC92 normalisation indicated that the variance in ERCC spike-ins was greater than in total RNA content of the samples. Therefore, we continued with murine mRNA size factor-normalised counts. Principal component analysis showed an extreme outlier (the third replicate of the 15-hour time point in the auxin data set), which was therefore excluded from further analyses. P values were computed on total or new RNA using the Wald test implemented in DESeq2, and fold changes were estimated using the PsiLFC estimator from the lfc package.

### RNA-seq

Cells were treated with 1 µM CB-6644 for 24 hours. RNA was extracted using the miRNeasy kit (Qiagen). RNA integrity was verified using the Standard Sensitivity RNA kit (Agilent Technologies) on a Fragment Analyzer system (Agilent Technologies). Samples that passed quality checks were processed by depleting rRNA with the NEBNext rRNA depletion kit v2 (NEB) and subsequent library preparation using the NEBNext Ultra II Directional RNA Library Prep kit (NEB). Libraries were sequenced for 2×60 cycles on a NextSeq2000 platform (Illumina). FASTQ files were aligned to the mm39 genome using STAR V.2.5.3a. Gene-level reads (Ensembl version 111) were counted using the GenomicAlignments R package (version 1.38.2) and differential gene expression analysis was performed with edgeR (V.4.0.16).

### Chromatin immunoprecipitation

For each immunoprecipitation sample, 50 million cells were crosslinked with formaldehyde (final concentration, 1%) for 10 min at room temperature, as described. Glycine was added to a final concentration of 125 mM to stop the crosslinking, and samples were incubated for 5 min at room temperature. Cells were washed twice with ice-cold PBS and resuspended in PBS supplemented with protease and phosphatase inhibitors (phosphatase inhibitor cocktail 2/3, P5726, P0044; protease inhibitor cocktail, P8340, Sigma-Aldrich). Buffers used in further steps were freshly supplemented with protease and phosphatase inhibitors.

To control for overall changes in chromatin binding, human or mouse cell chromatin was added to samples of KPC or A375 cells, respectively. In particular, 3 million U2OS osteosarcoma cells or NIH3T3 fibroblasts (6% of the starting cell number) were added to each sample. Then, samples were lysed in lysis buffer I (5 mM PIPES pH 8.0, 85 mM KCl, 0.5% NP-40) at 4°C for 20 min. Nuclei were collected by centrifugation (1500 rpm for 15 min at 4°C), and the pellets were dissolved in lysis buffer II (10 mM Tris pH 7.5, 150 mM NaCl, 1 mM EDTA, 1% NP-40, 1% sodium deoxycholate, 0.1% SDS). Crosslinked chromatin was fragmented by sonication (total duration 16 min with 10 s pulses and 45 s pauses). A fragment size distribution of 150–300 bp was verified by agarose gel electrophoresis. The samples were centrifuged (20 min at 14 000 rpm at 4°C), and the supernatant was taken as the sheared chromatin input for immunoprecipitation.

For immunoprecipitation, 100 µL Dynabeads Protein A and Protein G (Thermo Fisher Scientific) were preincubated overnight with 10 μg primary antibody in 5 g/L BSA in PBS. The antibodies were against RUVBL1 (Cell Signaling, 74775) and MYC (Abcam, ab32072). IgG (I5381, Sigma-Aldrich) was used as an isotype control. The antibody-coupled beads were washed three times with 5 g/L BSA in PBS. Sheared chromatin corresponding to 50 million cells was added and incubated with rotating for 6 hours at 4°C. Then, the beads were washed thrice with washing buffer I (20 mM Tris pH 8.1, 150 mM NaCl, 2 mM EDTA, 1% Triton X-100, 0.1% SDS), washing buffer II (20 mM Tris pH 8.1, 500 mM NaCl, 2 mM EDTA, 1% Triton X-100, 0.1% SDS), washing buffer III (10 mM Tris pH 8.1, 250 mM LiCl, 1 mM EDTA, 1% NP-40, 1% sodium deoxycholate; including a 5 min incubation step with rotation) and once with TE buffer (Thermo Fisher Scientific). Chromatin–protein complexes were eluted twice from the beads by incubating with 150 µL freshly prepared elution buffer (100 mM NaHCO_3_, 1% SDS) for 15 min at room temperature, with rotation. Decrosslinking of the eluted and input samples was done overnight, followed by digestion with proteinase K (Carl Roth) and RNase A (final concentration, 60 µg/mL). The DNA was purified by phenol-chloroform extraction and precipitated with ethanol. The resulting ChIP DNA pellets were dissolved in water for analysis.

### ChIP-qPCR and ChIP-seq

To assess the efficiency of immunoprecipitation, ChIP DNA pellets were analysed by qPCR on a StepOnePlus Real-Time PCR System (Thermo Fisher Scientific) using the SYBR Green Master Mix (Thermo Fisher Scientific). Equal amounts of ChIP DNA and SYBR Green Master Mix were added along with 0.5 μM primers. qPCR assays were done in technical triplicates.

For ChIP-seq, qPCR-verified ChIP DNA was quantified using the Quant-iT PicoGreen dsDNA assay (Thermo Fisher Scientific). Library preparation was done using the NEBNext Ultra II DNA Library Prep Kit for Illumina (NEB). The libraries were amplified using 13 PCR cycles. The concentration and size distribution of the library were evaluated on a Fragment Analyzer (Agilent Technologies) using the NGS Fragment High Sensitivity Analysis Kit (1–6000 bp; Agilent Technologies). The libraries were sequenced on a NextSeq500 platform for 75 cycles or a NextSeq2000 sequencer for 120 cycles (Illumina).

FASTQ files of input samples from auxin and DMSO-treated cells were combined. FASTQ files were aligned to the mm10 and hg19 genomes using Bowtie 2 V.2.3.4.1. BAM files were normalised by a scaling factor determined from the number of human or mouse reads per data set, respectively. Normalised BAM files were sorted using SAMtools version 1.7 and converted into bedgraphs with bedtools version 2.26.0. Coverage in promoter regions (transcription start site (TSS)±3 kb) was calculated using bedtools coverage on all annotated Ensembl genes (release 102). For MYC and RUVBL1, ChIP-seq peaks were called using MACS2 version 2.2.7.1 with p value cut-offs of 0.01 and 0.001, respectively. Promoter peaks were defined as peaks overlapping with promoters (TSS±3 kb). Overlap between MYC and RUVBL1 peaks and genomic feature annotations were calculated using ChIPpeakAnno version 3.30.1 and compared with the distribution of 1 million random 300 bp intervals. To plot heatmaps, BAM files were converted to bigWig files using deepTools version 3.5.1, and a read matrix was calculated from bigWig files around the peak positions of RUVBL1, MYC and the shared sites.

### Overexpression of MYC-ER and RUVBL1 mutants

To overexpress exogenous MYC, KPC cells were transduced via lentiviral integration with pRRLSin.cPPT.SFFV-IRES-Puro.WPRE containing a MYC-ER insert. To overexpress RUVBL1, KPC cells were transduced with pRRLSin.cPPT.SFFV-IRES-Puro.WPRE containing a RUVBL1^WT^, RUVBL1^A62T^, RUVBL1^D302N^, RUVBL1^A62T, D302N^, RUVBL1^Δ94-118^, RUVBL1^Δ102-107^ or RUVBL1^K108A^ open reading frame via lentiviral integration. RUVBL1 mutants were overexpressed in KPC or KPC*^AID-Ruvbl1; TIR1^* cells by lentiviral transduction with pRRLSin.cPPT.SFFV-IRES-Puro.WPRE, respectively. Cells were selected with 2 µg/mL puromycin (InvivoGen) for 3 days.

### *E. coli* expression and purification of MYC^1-163^ and RUVBL1/2

Human MYC (1-163) was cloned into a modified pET28b vector following an N-terminal Hisx6-Maltose binding protein (MBP) tag and a tobacco etch virus (TEV) protease cleavage site (Addgene: 29654). Protein was expressed in the Bl21 (DE3) RIL *E. coli* strain at 37°C. Protein expression was induced by adding 0.5 mM IPTG to the medium when the culture reached an optical density of 0.5 and letting the culture grow for an additional 3 hours at 37°C. Protein purification steps were performed at 4°C.

The cells were collected by centrifugation and resuspended in lysis buffer (20 mM Tris-HCl pH 7.9, 30 mM imidazole, 500 mM NaCl, 10% glycerol, 5 mM beta-mercaptoethanol, 0.284 µg/mL leupeptin, 1.37 µg/mL pepstatin A, 0.17 mg/mL PMSF, 0.33 mg/mL benzamidine). Cells were lysed by sonication, and lysates were clarified by centrifugation. Clarified lysate was applied to a 5 mL HisTrap column equilibrated in lysis buffer. The column was washed with lysis buffer and additionally with five column volumes of a high salt buffer (lysis buffer with 1 M NaCl) before returning to lysis buffer. Protein was eluted from the HisTrap column with elution buffer (20 mM Tris-HCl pH 7.9, 500 mM imidazole pH 8.0, 500 mM NaCl, 10% glycerol, 5 mM beta-mercaptoethanol) and loaded directly onto a 10 mL amylose column (New England Biolabs). The amylose column was washed with lysis buffer, and bound protein was eluted with a maltose-containing buffer (20 mM Tris-HCl pH 7.9, 30 mM imidazole pH 8.0, 500 mM NaCl, 116 mM maltose, 10% glycerol, 5 mM beta-mercaptoethanol). Fractions containing MYC, identified by SDS-PAGE and Coomassie staining, were pooled and concentrated on an Amicon Millipore ultrafiltration device (10 000 molecular weight cut-off). Concentrated protein was applied to a HiLoad Superdex 200 16/600pg column (GE Healthcare) equilibrated in size exclusion buffer (20 mM Tris-HCl pH 7.9, 500 mM NaCl, 10% glycerol, 1 mM DTT). Fractions containing MYC were pooled and concentrated again. Protein concentration was determined using the calculated extinction coefficient for His6-MBP-MYC and the absorbance at 280 nm. Protein was aliquoted, snap frozen in liquid nitrogen and stored at −80°C until use.

Full-length human RUVBL1 and RUVBL2 were cloned into vectors 14B (Addgene: 48308) and 14A (Addgene: 48307), respectively, via ligation-independent cloning. 14B contains a Hisx6 tag followed by a TEV cleavage site. The vectors were combined to create a co-expression vector. The proteins were expressed via autoinduction in BL21 (DE) RIL LOBSTR *E. coli*. Protein purification steps were performed at 4°C unless otherwise noted.

Cells were collected by centrifugation and resuspended in buffer A (20 mM Tris-HCl pH 7.9, 30 mM imidazole pH 8.0, 300 mM NaCl, 1 mM MgCl_2_, 10% glycerol, 5 mM beta-mercaptoethanol, 0.284 µg/mL leupeptin, 1.37 µg/mL pepstatin A, 0.17 mg/mL PMSF, 0.33 mg/mL benzamidine). Cells were lysed by sonication, and lysates were clarified by centrifugation. Clarified lysate was applied to a 5 mL HisTrap column equilibrated in buffer A. The column was washed with buffer A and with five column volumes of a high salt buffer (lysis buffer as above with 1 M NaCl) before returning to lysis buffer and then low salt buffer (lysis buffer with 150 mM NaCl). Protein was eluted from the HisTrap column with elution buffer (20 mM Tris-HCl pH 7.9, 500 mM imidazole pH 8.0, 150 mM NaCl, 1 mM MgCl_2_, 10% glycerol, 5 mM beta-mercaptoethanol) and loaded directly onto a 5 mL HiTrap Q column (GE Healthcare) equilibrated in low salt buffer. The HiTrap Q column was washed with low salt buffer, and bound proteins were eluted with a linear gradient (30 min, 1.5 mL/min) into 100% high salt buffer. Fractions were evaluated by SDS-PAGE and Coomassie staining, and those containing RUVBL1/2 were pooled and mixed with 1.5 mg His6-TEV protease. The protein was dialysed against 1 L lysis buffer overnight and then applied to a 5 mL HisTrap column equilibrated in lysis buffer to remove uncleaved protein and TEV protease. The follow-through was collected and concentrated with an Amicon Millipore ultrafiltration device (30 000 molecular weight cut-off). The concentrated protein was applied to a Superose 6 Increase 10/300 column (GE Healthcare) equilibrated in size exclusion buffer (20 mM Tris-HCl pH 7.9, 200 mM NaCl, 1 mM MgCl_2_, 10% glycerol, 5 mM beta-mercaptoethanol). Eluted protein was identified by SDS-PAGE and Coomassie staining, and fractions containing RUVBL1 and RUVBL2 were pooled and concentrated again. Protein concentration was determined using the calculated extinction coefficient for RUVBL1/2 and the absorbance at 280 nm. Protein was aliquoted, snap frozen in liquid nitrogen and stored at −80°C until use.

### Gradient centrifugation

For the complex formation assay, purified RUVBL1/2 (20 µM), MYC (20 µM) or both were mixed with ADP-BeF (1 mM) in 20 mM Na·HEPES pH 7.4, 50 mM NaCl, 1 mM DTT, 3 mM MgCl_2_. The solution (100 µL) was applied to a 10–30% sucrose gradient and centrifuged for 16 hours at 32 000 rpm at 4°C in an SW41 rotor (Beckman). Then, 200 µL samples were sequentially fractionated from the top of the gradient. 15 µL of each sample was analysed by 10% SDS-PAGE, and proteins were identified by Coomassie blue staining.

### Pull-down assay

His6-MBP-MYC^1-163^ (5 µM) was incubated with 15 µM of the full-length RUVBL1/2 complex in a final assay buffer containing 50 mM NaCl, 20 mM Tris-HCl pH 7.9, 3 mM MgCl_2_, 1 mM DTT and 10% glycerol (final volume 10 µL). The protein was then added to 50 µL of amylose beads (New England Biolabs) that were equilibrated in the assay buffer. The complexes were incubated for 20 min at room temperature in a thermomixer (300 rpm). The beads were then washed three times with 500 µL assay buffer. After the final wash, protein was eluted from the beads with 30 µL of assay buffer supplemented with 116 mM maltose. The eluted protein was applied to a 10% SDS-PAGE and proteins were visualised using Coomassie blue.

### Immunohistochemistry

After explantation, tumours were fixed in 4% formaldehyde overnight and washed with 70% ethanol. They were dehydrated by subsequent immersing in increasing concentrations of ethanol and finally xylol (Carl Roth). Tumours were embedded in paraffin, and tissue sections were cut and placed on slides. Sections were deparaffinised by immersing in xylol and rehydrated by immersing in decreasing concentrations of ethanol. Antigens were retrieved by boiling the slides in 10 mM sodium citrate buffer pH 6 for 15 min. Peroxidases were blocked by treating for 10 min with 3% H_2_O_2_. Sections were washed twice in TBS and blocked for 1 hour at room temperature with 10% goat serum (G6767, Sigma-Aldrich) in TBS. Sections were incubated with a primary antibody (RUVBL1, Cell Signaling, 74775S, 1:100; KI67, RM-9106-S, 1:200; BrdU, Bio-Rad, OBT0030G, 1:200; CD3, Proteintech, 17617-1-AP, 1:10 000) in 5% goat serum in TBS overnight at 4°C. Slides were washed thrice with TBS, once again blocked in 10% goat serum in TBS, and incubated with an HRP-conjugated secondary antibody (anti-rabbit IgG: Thermo Fisher Scientific, B40962; anti-rat IgG: Sigma-Aldrich, GENA935) for 1 hour at room temperature. Slides were washed thrice with TBS and signals were developed using the SignalStain DAB Substrate kit (8059, Cell Signaling). Slides were counterstained with haematoxylin solution (GHS332, Sigma-Aldrich) and dehydrated in increasing concentrations of ethanol and finally xylol before mounting them with Cytoseal 60 mounting medium (Thermo Fisher Scientific) and letting them dry. Slides were scanned using a Pannoramic Desk slide scanner (3DHISTECH), and images were analysed using QuPath software V.0.3.2. For the CD3 staining of tumours induced by 24031 or 9172 cells treated with CB-6644 or vehicle, similar size (small) tumour lesions (24031: 8200–12 000 cells, 9172: 380–1600 cells) were compared. The human TMA representing primary PDAC tissue and tumour stroma from 31 individuals and benign pancreatic tissue, that is, acinar and ductal tissue, from 24 individuals who underwent surgery for PDAC at the university hospital in Würzburg, Germany between 2010 and 2020 was stained for RUVBL1 as described above. Histoscore distributions were compared with the Wilcoxon signed-rank test.

### Liquid chromatography–MS quantification of CB-6644 from tissue

For the analysis of CB-6644 in tissues, samples were homogenised in 19 volumes of methanol/water (80/20, v/v) in Eppendorf tubes using a potter elvehjem homogenisator equipped with a stainless steel pistil (10 strokes at 1200 rpm). 200 µL of the resulting homogenate or, in case of blood samples, 10 µL sample in 200 µL methanol/water (80/20, v/v) was diluted with 628 µL of 0.01 µM lamivudine in methanol/water (80/20, v/v), centrifuged (2 min maximum rpm) and the resulting supernatant was applied to activated (with 280 µL acetonitrile) and equilibrated (with 280 µL methanol/H_2_O (80/20, v/v)) C18-SPE columns (Phenomenex Strata C18-E (50 mg) (Aschaffenburg, Germany)). Another 180 µL ethanol/H_2_O (80/20, v/v) was applied to the column and the eluates were collected in an Eppendorf tube and evaporated to dryness in a rotary vacuum concentrator (speed vac). Prior analysis, the dry residues were redissolved in 75 µL of 5 mM NH4OAc in acetonitrile/H_2_O (50/50, v/v). Following centrifugation for 2 min at maximum rpm, the supernatant was transferred onto autosampler glass vials and stored at 15°C for further analysis.

Liquid chromatography (LC)–MS analysis was performed using a Q Exactive mass spectrometer coupled to a Dionex U3000 UHPLC system (Thermo Fisher Scientific). The mass spectrometer was operated in full MS positive mode applying the following MS parameters: scan range, 69–1000 m/z; resolution, 70 000; automatic gain control target, 3E6; maximum injection time, 200 ms; sheath gas, 30; auxiliary gas, 10; sweep gas, 3; spray voltage, 3.6 kV; capillary temperature, 320°C; S-lens radio frequency level, 55.0; auxiliary gas heater temperature, 120°C. The LC system was fitted with an Accucore Biphenyl column (2.6 μm particles, 100×2.1 mm) (Thermo Scientific, Bremen, Germany) and particle filter (Supelco ColumnSaver 0.5 µm (Merck, Darmstadt, Germany; 55214-U)). The column temperature was maintained at 45°C. The mobile phase was composed of 5 mM NH4OAc in acetonitrile/H_2_O (5/95, v/v) (solvent A) and 5 mM NH4OAc in acetonitrile/H_2_O (95/5, v/v) (solvent B). Compounds were eluted at a flow rate of 0.2 mL/min, applying a gradient of 10% solvent B for 2 min, followed by a linear decrease to 100% solvent B within 8 min, then maintaining 100% solvent B for 9 min, then returning to 10% solvent B in 1 min, and 5 min 10% solvent B for column equilibration before each injection.

Annotation and data evaluation: peaks corresponding to the calculated monoisotopic masses (MIM±2 mMU) were integrated using TraceFinder software (V.3.3.350.0; Thermo Fisher Scientific). Absolute quantification of compounds was performed by interpolation of the corresponding standard curves obtained from commercially available compounds running with the same batch of samples.

## Data Availability

Data are available in a public, open access repository. Primary sequencing data (shRNA screen, SLAM-seq, ChIP-seq, RNA-seq) have been deposited in the Gene Expression Omnibus under the accession number GSE216095. All other data relevant to the study are included in the article or uploaded as supplementary information.
