# Supplementary material for: Targeting MYC effector functions in pancreatic cancer by inhibiting the ATPase RUVBL1/2
Source: Gut. 2024 May 31;73(9):1509–28. doi: 10.1136/gutjnl-2023-331519 (PMC11347226; doi:10.1136/gutjnl-2023-331519)
Supplement: Supplementary data [file gutjnl-2023-331519supp002.pdf]

Online supplemental figure S1

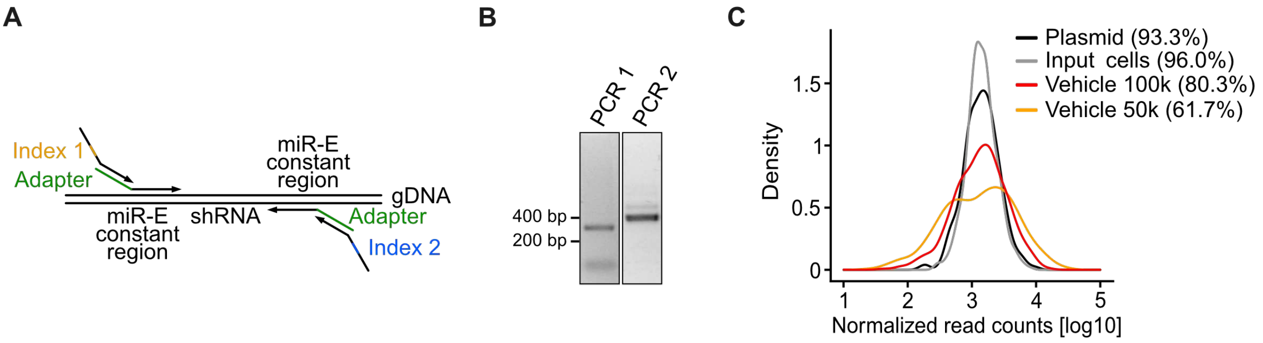

Online supplemental figure S1. Genetic dropout screens reveal differential dependence on MYC binding partners in PDAC *in vitro* and *in vivo* (related to figure 1)

(A) Strategy for sequencing the shRNA library from plasmid DNA or genomic DNA (gDNA). Arrows indicate primers.

(B) Agarose gel pictures of the two-step library preparation procedure of the shRNA miR-E backbone before sequencing. PCR 1 introduced adapters, and PCR 2 introduced dual barcodes for multiplexing.

(C) Density plots of the shRNA distribution in different conditions: *Plasmid*, plasmid library; *Input cells*, KPC cells transduced with the shRNA library, which were implanted for the *in vivo* screen; *Vehicle 100k*, tumors harvested 14 days after the injection of 100,000 transduced KPC cells; *Vehicle 50k*, tumors harvested 14 days after the injection of 50,000 transduced KPC cells. The percentage of shRNAs that differ less than 10-fold are indicated.

Online supplemental figure S2

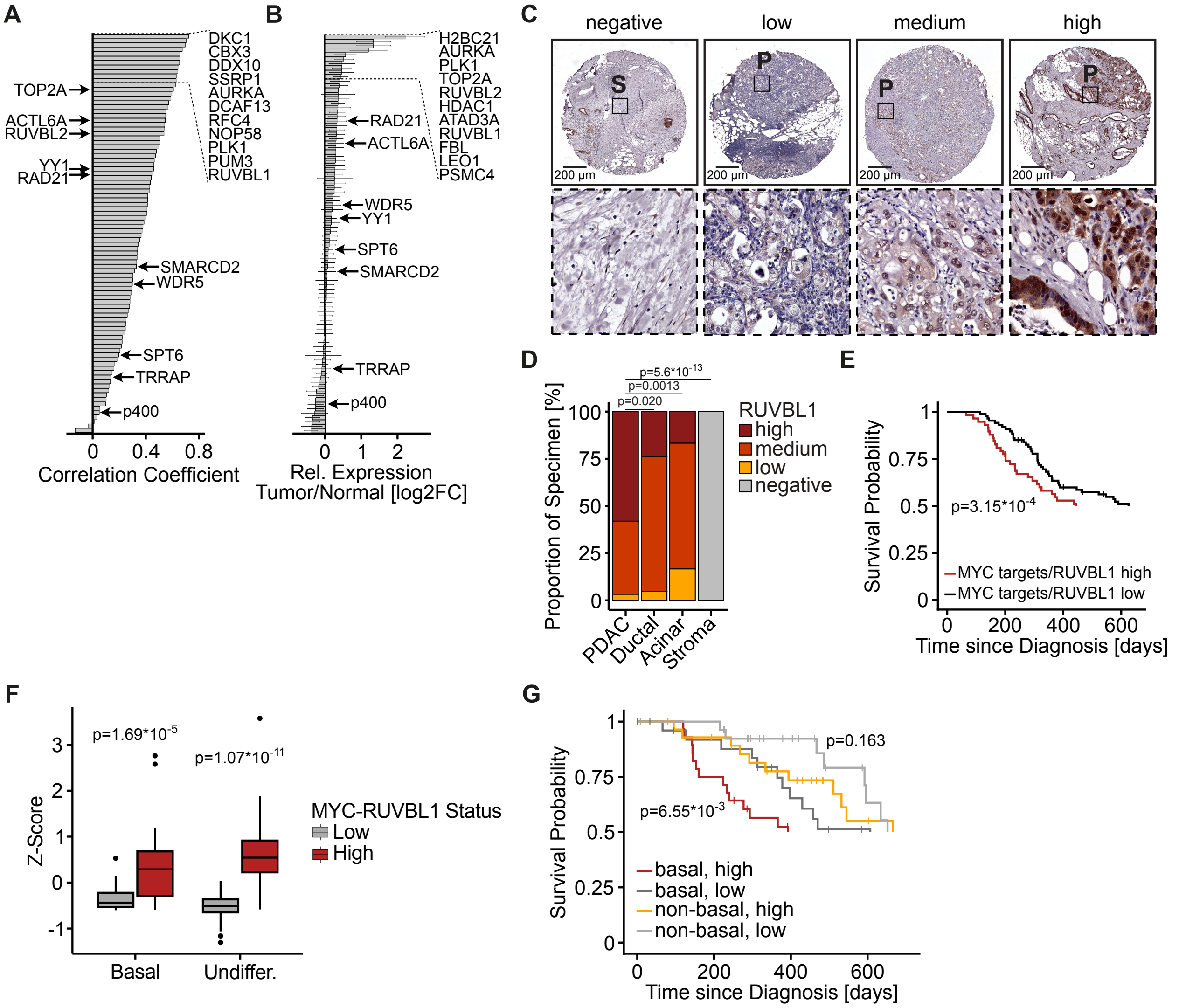

Online supplemental figure S2. Expression of MYC and RUVBL1 correlate in PDAC and high levels are associated with aggressive tumors (related to figure 2)

(A) Bar graph showing the Pearson correlation coefficient of RNA expression of all 91 MYC binding partners and MYC target gene expression (mean of all HALLMARK MYC TARGET V1 genes after scaling expression (FPKM) across all TCGA patients) in PDAC tumors (n=159).

(B) Bar graph of differential gene expression analysis of the 91 MYC binding partners in primary tumors (n=159) compared to adjacent healthy tissue (n=4) of PDAC patients in the TCGA database. Values are log2FC ± SEM.

(C) Representative images of RUVBL1 IHC staining of the human PDAC tissue microarray. The top row shows representative cores of PDAC and the bottom row zoomed magnifications of the area marked with a dashed box of these cores with the associated histoscore. Scale bar represents 200 µm. The panel with RUVBL1 high score is also shown in figure 2B. S, stroma; P, PDAC.

(D) Distribution of histoscores from panel C in 31 PDAC samples (PDAC and Stroma) and 24 benign pancreatic tissue samples (Acinar, Ductal). p-value, Wilcoxon signed-rank test.

(E) Kaplan-Meier survival curves for PDAC patients (ICGC, PACA-CA) stratified into groups of low and high expression of RUVBL1 and MYC target genes (mean of all MYC TARGET V1 genes after scaling expression (FPKM) across all patients). p-value, Log-rank test.

(F) Boxplot of basal-like PDAC and differentiation scores in RUVBL1/MYC-high and low tumors. Scores represent the mean expression of all genes in the gene set (Composition: Basal-like subtype WB5390, Differentiation: RHODES_UNDIFFERENTIATED_CANCER, MSigDB) after scaling expression (FPKM) across all TCGA PDAC patients. p-value, unpaired *t*-test.

(G) Kaplan-Meier survival curves for TCGA PDAC patients stratified into groups of low and high expression of RUVBL1 and MYC target genes, and low and high expression of markers for basal-like PDAC (Basal-like subtype WP5390) and undifferentiated cancer (RHODES_UNDIFFERENTIATED_CANCER, MSigDB). Scores represent the mean expression of all genes in the gene set after scaling expression (FPKM) across all patients.

Online supplemental figure S3

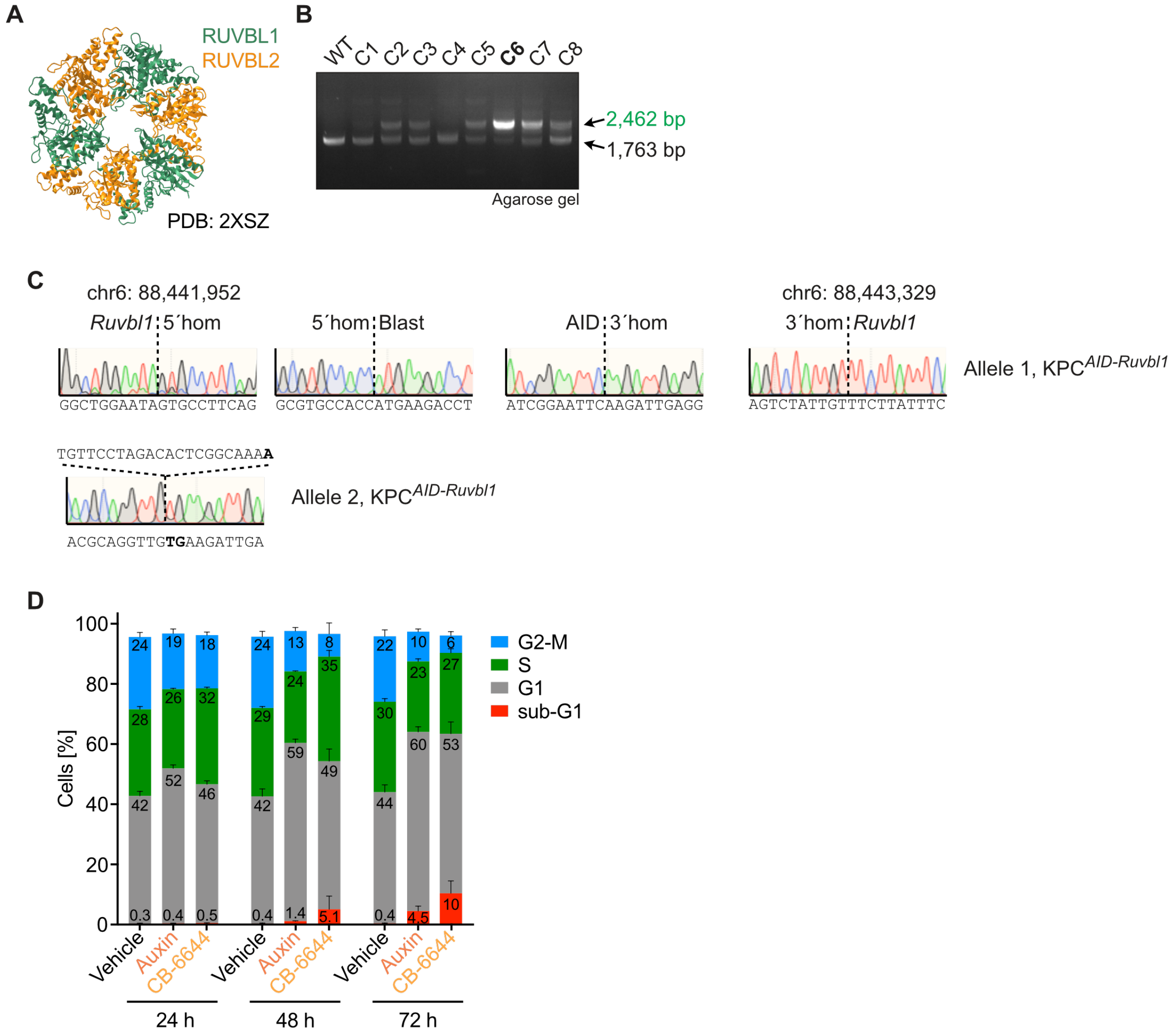

Online supplemental figure S3. RUVBL1 is essential for DNA replication and growth of pancreatic cancer cells (related to figure 3)

(A) X-ray crystal structure (PDB: 2XSZ) of the hexameric RUVBL1/2 complex.

(B) Agarose gel of genotyping PCR, as indicated in figure 3A, of eight AID-*Ruvbl1* knock-in clones and wild-type (WT) KPC cells. Amplicon sizes for the WT and genome-edited *Ruvbl1* loci are indicated.

(C) Sanger sequencing results for both *Ruvbl1* alleles of clone C6. Borders between the genomic sequence and homology arms as well as between the homology arms and insert are shown. The deleted start codon of the second allele is marked in bold.

(D) PI cell cycle profile of KPC$^{AID\text{-}Ruvbl1;\ TIR1}$ cells treated with DMSO (Vehicle), auxin or CB-6644 for 24, 48 and 72 h. The experiment was performed in biological triplicates (mean ± SD).

Online supplemental figure S4

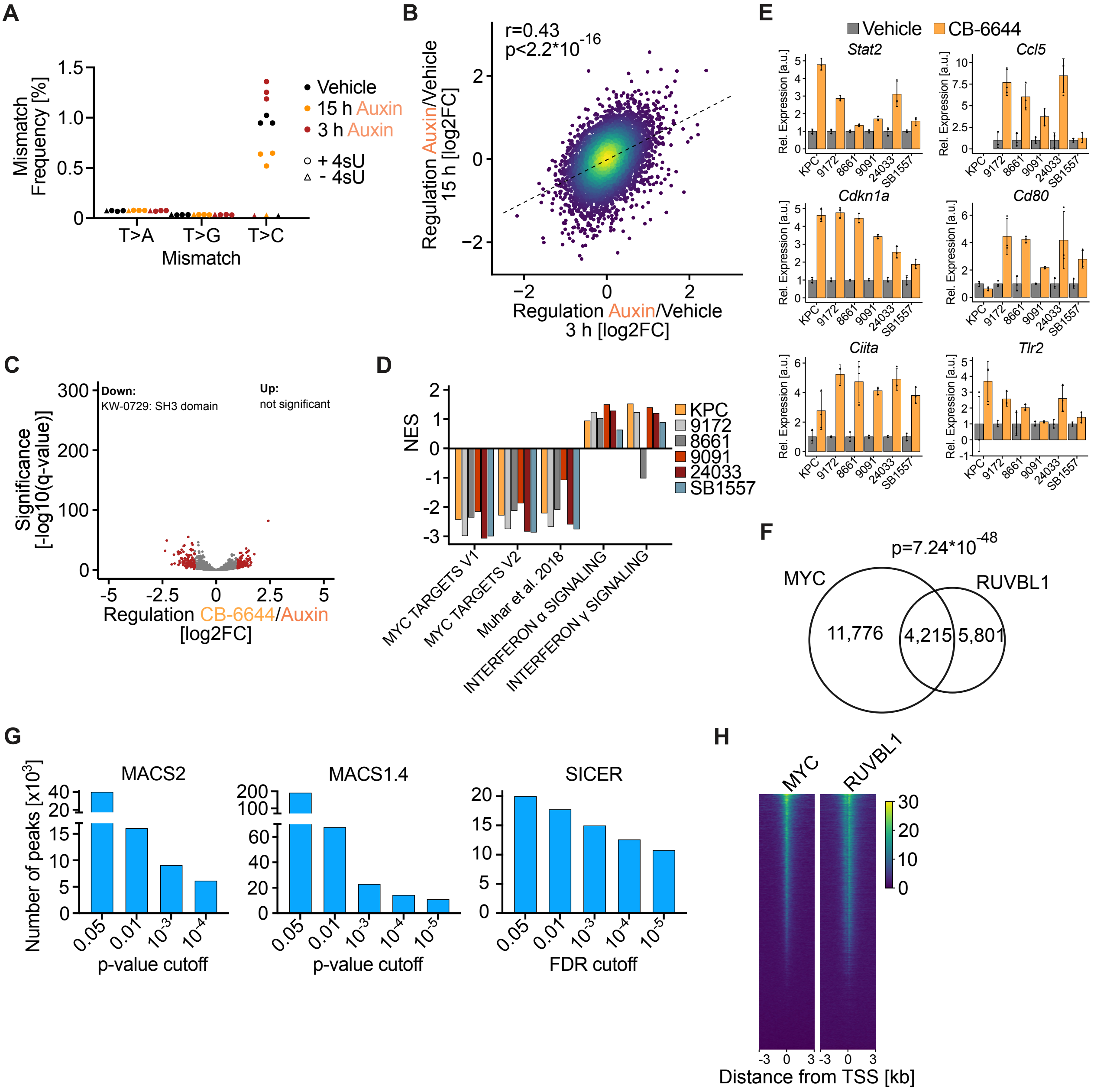

Online supplemental figure S4. RUVBL1 redirects transcription from immune genes to growth genes (related to figure 4)

(A) Jitter plot of mismatch frequency of thymine in SLAM-seq experiments. KPC$^{AID\text{-}Ruvbl1;\ TIR1}$ cells were treated with auxin for 3 or 15 h followed by 2 h of labeling with 4sU. Alkylation of 4sU introduces T>C conversions.

(B) Scatter plot comparing gene regulation after targeted degradation of RUVBL1 for 3 h and 15 h. KPC$^{AID\text{-}Ruvbl1;\ TIR1}$ cells were treated with 1 µM auxin for 3 or 15 h (n=3). Gene expression was analyzed by SLAM-seq. Changes (log2FC) in 4sU-labeled RNA vs. DMSO (Vehicle)-treated cells are shown (r, Pearson's correlation coefficient; p, unpaired *t*-test).

(C) Volcano plot of SLAM-seq data comparing RUVBL1 depletion and RUVBL1/2 inhibition. KPC$^{AID\text{-}Ruvbl1;\ TIR1}$ or KPC$^{MYC\text{-}ER}$ cells were treated with 1 µM auxin for 15 or 1 µM CB-6644 for 24 h respectively. q-value, FDR corrected Wald-test.

(D) GSEA results of RNA-seq data of five additional murine PDAC cell lines with differing genotypes representing different PDAC subtypes compared to SLAM-seq data of KPC cells. Cells were treated with 1 µM CB-6644 or DMSO for 24 h. A positive normalized enrichment score (NES) depicts gene sets activated after RUVBL1/2 inhibition.

(E) Bar plots of selected immune genes and their regulation (RNA-seq) upon 24 h CB-6644 treatment in a murine PDAC cell panel as in panel D.

(F) Venn diagram of RUVBL1 and MYC peaks of chromatin binding. The p-value was calculated using a hypergeometric test assuming a total of 44,522 nonoverlapping promoters.

(G) Bar plots showing the number of identified MYC peaks in KPC$^{AID\text{-}Ruvbl1;\ TIR1}$ cells by different peak calling algorithms and cutoffs.

(H) Heatmap of RUVBL1 and MYC chromatin binding at promoters, estimated in vehicle treated KPC$^{AID\text{-}Ruvbl1;\ TIR1}$ cells. Binding is expressed as spike-normalized reads. Genes are sorted according to the amount of MYC bound to their promoter.

Online supplemental figure S5

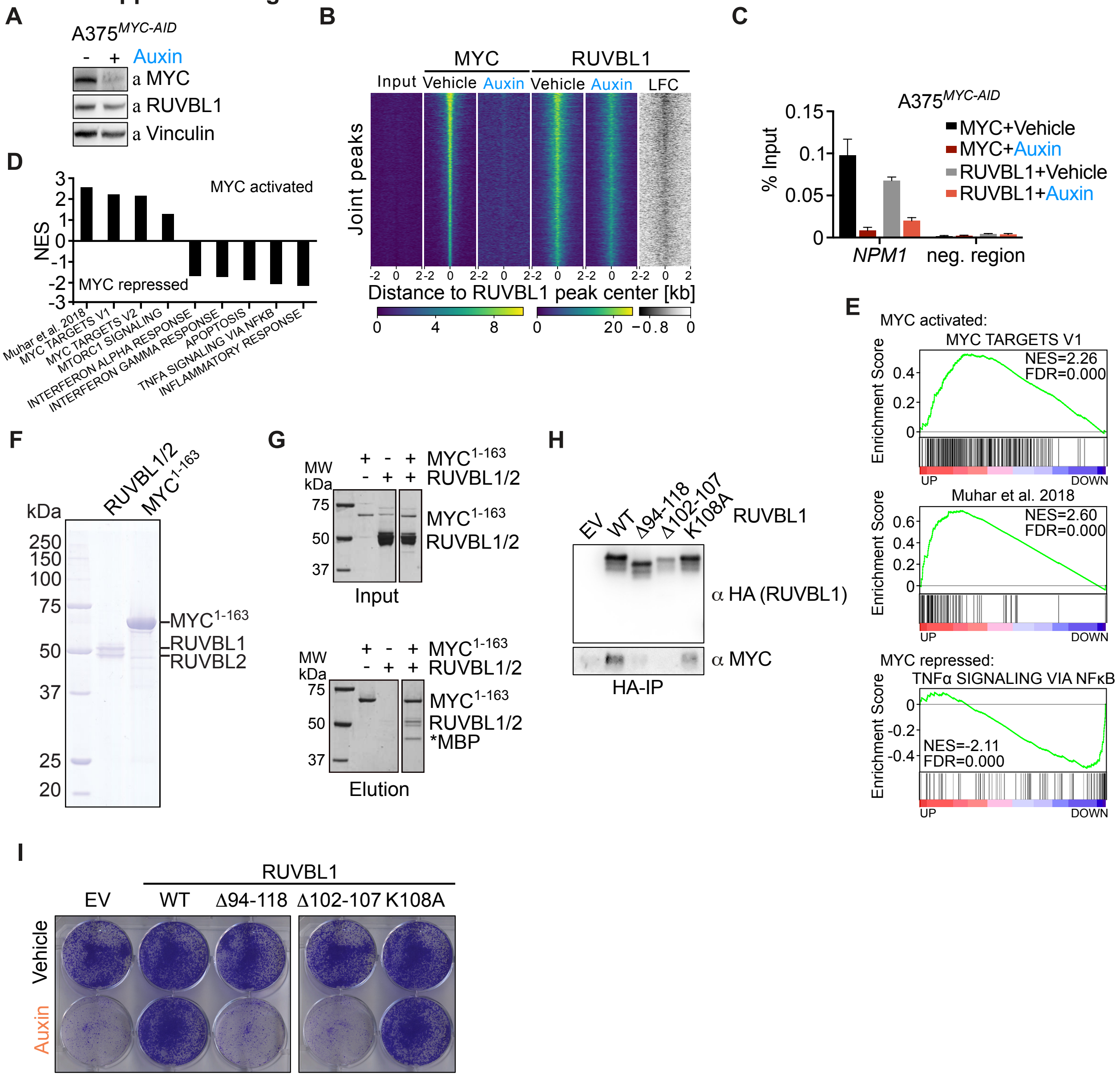

Online supplemental figure S5. RUVBL is an essential cofactor of MYC (related to figure 5)

(A) Immunoblot of RUVBL1 and MYC in A375$^{MYC\text{-}AID}$ cells treated with DMSO or 1 µM auxin for 3 h. Vinculin, loading control.

(B) Heatmap of MYC and RUVBL1 chromatin binding around MYC-RUVBL1 joint peaks after MYC depletion. A375$^{MYC\text{-}AID}$ cells were treated with auxin for 3 h. Binding is shown as spike-normalized reads. LFC, log2(auxin/vehicle).

(C) ChIP-qPCR of RUVBL1 at promoter regions in A375$^{MYC\text{-}AID}$ cells treated with 1 µM auxin for 3 h or vehicle control. Binding was assessed in the promoter of the *NPM1* gene or in a gene-free genomic region (neg. region).

(D) Bar graph of GSEA results of SLAM-seq data from KPC$^{MYC\text{-}ER}$ cells. Cells were treated with 200 nM 4-hydroxytamoxifen or ethanol for 4 h, followed by 400 µM 4sU for 2 h. A positive normalized enrichment score (NES) depicts gene sets activated by MYC.

(E) GSEA enrichment plots of selected MYC-activated and MYC-repressed gene sets. GSEA was performed on SLAM-seq data from KPC$^{MYC\text{-}ER}$ cells treated with 200 nM 4-hydroxytamoxifen or ethanol for 4 h followed by

400 μM 4sU for 2 h. A positive normalized enrichment score (NES) depicts gene sets activated by MYC (FDR, false discovery rate).

(F) Coomassie blue-stained gel (10% SDS-PAGE) showing the purity of recombinant RUVBL1/2 and His6-MBP-MYC^{1-163} (0.5 μg per lane).

(G) Pull-down experiment with purified 6xHis-MBP-MYC^{1-163} (MYC^{1-163}, 5 μM) and RUVBL1/2 (15 μM). Proteins were incubated with amylose resin and eluted using a maltose containing buffer. Proteins eluted from the amylose beads were applied to 10% SDS-PAGE and stained with Coomassie Blue.

(H) RUVBL1 and MYC immunoblot after HA-IP of RUVBL1 mutants. $KPC^{AID\text{-}Ruvbl1;\ TIR1}$ cells were transduced with the indicated HA-tagged RUVBL1-mutants. Cells were treated with auxin for 3 h to deplete endogenous AID-RUVBL1. HA-RUVBL1 mutants were immunoprecipitated and the eluate was analyzed by immunoblot and probed with an HA and MYC antibody.

(I) Crystal violet staining of $KPC^{AID\text{-}Ruvbl1;\ TIR1}$ cells overexpressing the indicated RUVBL1 mutants as in panel H. Cells were treated with auxin or vehicle for 5 days and viability was visualized by crystal violet.

Online supplemental figure S6

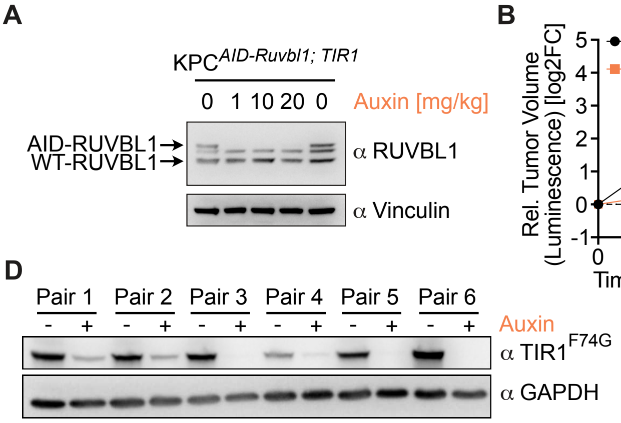

Online supplemental figure S6. RUVBL1 is required for the maintenance and progression of pancreatic cancer (related to figure 6)

(A) Immunoblot of pancreatic tumor lysates. KPC$^{AID\text{-}Ruvbl1;\ TIR1}$ cells were transplanted into 4 mice. After 18 d, mice were treated with auxin or vehicle for 6 h. Lysates of tumors were analyzed using an anti-RUVBL1 antibody. The band between WT and AID-tagged RUVBL1 can be attributed to murine IgG in the tissue. Vinculin, loading control.

(B) Relative volumes of late-stage pancreatic tumors (i.e., allowed to grow for 16 days prior to treatment). Mice were treated daily with 20 mg/kg auxin (n=6) or vehicle (n=6). Data are mean ± SEM. * n=4.

(C) Kaplan-Meier survival curves of mice with late-stage pancreatic tumors as in panel B. Vehicle, n=6. Auxin, n=6. p-value, log-rank test.

(D) Immunoblot of TIR1^{F74G} in lysates of late-stage pancreatic tumors (as in panels B and C) excised when mice reached the humane endpoint. TIR1^{F74G} was detected with an antibody against the MYC tag. GAPDH, loading control.

Online supplemental figure S7

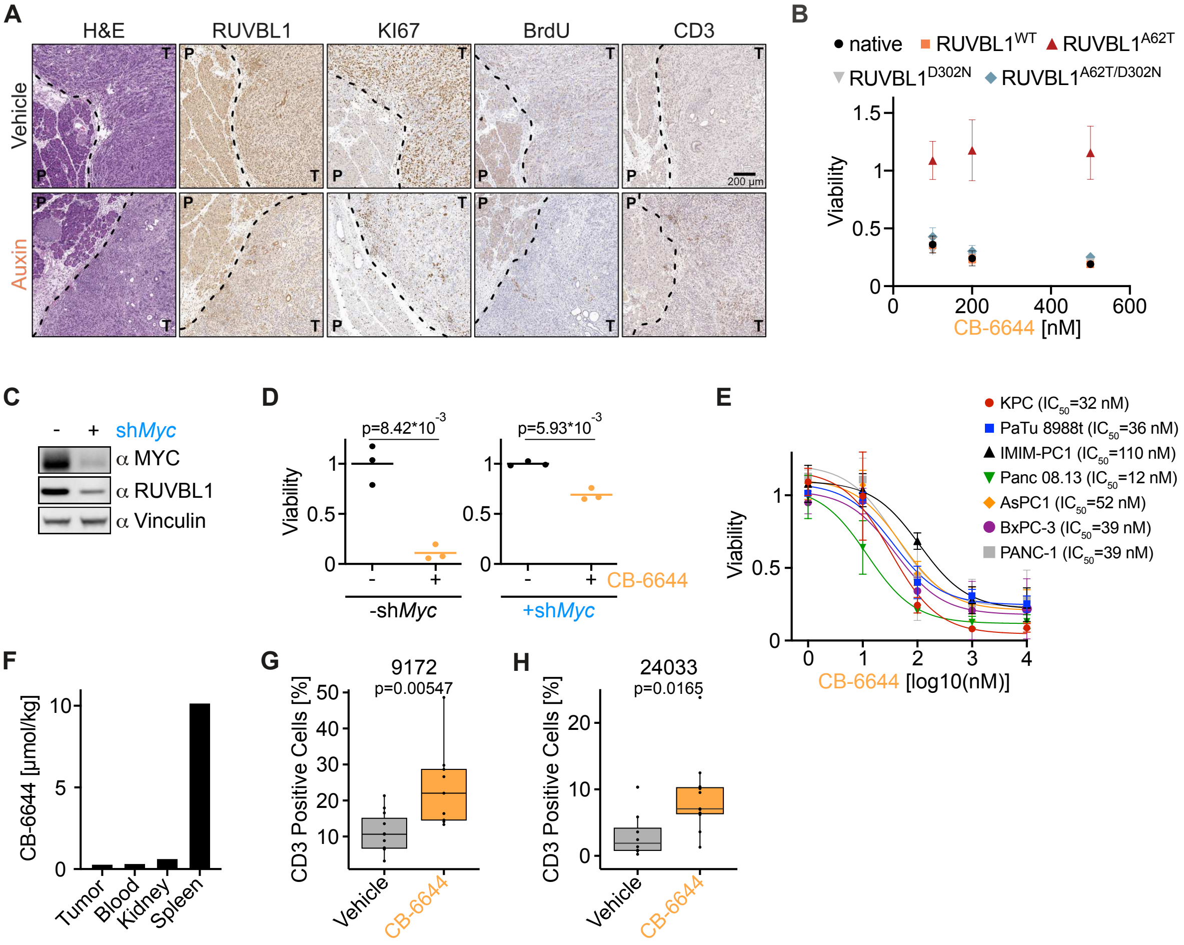

Online supplemental figure S7. RUVBL1 promotes immune evasion in PDAC (related to figure 7)

(A) H&E staining and immunohistochemical staining of RUVBL1, KI67, BrdU and CD3 on pancreatic sections containing KPC$^{AID\text{-}Ruvbl1;\ TIR1}$ tumors (T) and healthy pancreas (P) from mice treated with vehicle or 20 mg/kg auxin for 5 d.

(B) Viability (resazurin assay) of KPC cells overexpressing wild type, an inhibitor-resistant (A62T), catalytically dead (D302N) or double mutant (A62T, D302N) RUVBL1 treated with CB-6644 or vehicle for 72 h.

(C) Immunoblot of MYC and RUVBL1 in KPC^{shMyc} cells treated for 48 h with ethanol (-) or doxycycline (+) to induce sh*Myc* expression. Vinculin, loading control.

(D) Viability (resazurin assay) of KPC^{shMyc} cells treated for 48 h with ethanol (-sh*Myc*) or doxycycline (+sh*Myc*) to induce sh*Myc* expression and then treated with DMSO (vehicle; black dots) or 200 nM CB-6644 for 72 h. Unpaired t-test (n=3).

(E) Dose-response curves of CB-6644 on viability of human PDAC cell lines treated for 72 h. Cell viability was measured using the resazurin assay (n=3, mean ± SD).

(F) Quantification of CB-6644 concentrations in organs from a mouse with pancreatic tumors. The mouse was treated with 25 mg/kg CB-6644 twice daily for 2 days. Tissues were harvested on the third treatment day 16 h after the fifth injection, and CB-6644 levels were quantified using mass spectrometry.

(G) Quantification of immunohistochemical staining of CD3 in tumors formed by 9172 cells (*Kras*$^{G12D/+}$, Trp53$^{R172H/+}$). The cells were allowed to engraft for 7 days into 12 mice. Mice were treated with 25 mg/kg CB-6644 twice daily for 5 days. CD3-staining in similar sized tumor lesions was compared.

(H) Quantification of immunohistochemical staining of CD3 in tumors formed by 24031 cells (*Kras*$^{G12D/+}$, *Cdkn2a*$^{-/-}$). The cells were allowed to engraft for 7 days into 12 mice. Mice were treated with 25 mg/kg CB-6644 twice daily for 5 days. CD3-staining in similar sized tumor lesions was compared.
